# Comprehensive meta-analysis, co-expression, and miRNA nested network analysis identifies gene candidates in citrus against Huanglongbing disease

**DOI:** 10.1186/s12870-015-0568-4

**Published:** 2015-07-28

**Authors:** Nidhi Rawat, Sandhya P. Kiran, Dongliang Du, Fred G. Gmitter, Zhanao Deng

**Affiliations:** University of Florida, Institute of Food and Agricultural Sciences, Gulf Coast Research and Education Center, Wimauma, FL 33598 USA; Ocimum BioSolutions, Banjara Hills Road No. 1, VI Floor Reliance Classic, Hyderabad, 500039 India; University of Florida, Institute of Food and Agricultural Sciences, Citrus Research and Education Center, Lake Alfred, FL 33850 USA

**Keywords:** Citrus, Gene co-expression analysis, Gene ontology, Huanglongbing, HLB resistance, Meta-analysis, miRNA network analysis, Common and R-specific probe sets

## Abstract

**Background:**

Huanglongbing (HLB), the most devastating disease of citrus, is associated with infection by *Candidatus* Liberibacter asiaticus (*Ca*Las) and is vectored by the Asian citrus psyllid (ACP). Recently, the molecular basis of citrus–HLB interactions has been examined using transcriptome analyses, and these analyses have identified many probe sets and pathways modulated by *Ca*Las infection among different citrus cultivars. However, lack of consistency among reported findings indicates that an integrative approach is needed. This study was designed to identify the candidate probe sets in citrus–HLB interactions using meta-analysis and gene co-expression network modelling.

**Results:**

Twenty-two publically available transcriptome studies on citrus–HLB interactions, comprising 18 susceptible (S) datasets and four resistant (R) datasets, were investigated using Limma and RankProd methods of meta-analysis. A combined list of 7,412 differentially expressed probe sets was generated using a Teradata in-house Structured Query Language (SQL) script. We identified the 65 most common probe sets modulated in HLB disease among different tissues from the S and R datasets. Gene ontology analysis of these probe sets suggested that carbohydrate metabolism, nutrient transport, and biotic stress were the core pathways that were modulated in citrus by *Ca*Las infection and HLB development. We also identified R-specific probe sets, which encoded leucine-rich repeat proteins, chitinase, constitutive disease resistance (CDR), miraculins, and lectins. Weighted gene co-expression network analysis (WGCNA) was conducted on 3,499 probe sets, and 21 modules with major hub probe sets were identified. Further, a miRNA nested network was created to examine gene regulation of the 3,499 target probe sets. Results suggest that csi-miR167 and csi-miR396 could affect ion transporters and defence response pathways, respectively.

**Conclusion:**

Most of the potential candidate hub probe sets were co-expressed with gibberellin pathway (GA)-related probe sets, implying the role of GA signalling in HLB resistance. Our findings contribute to the integration of existing citrus–HLB transcriptome data that will help to elucidate the holistic picture of the citrus–HLB interaction. The citrus probe sets identified in this analysis signify a robust set of HLB-responsive candidates that are useful for further validation.

**Electronic supplementary material:**

The online version of this article (doi:10.1186/s12870-015-0568-4) contains supplementary material, which is available to authorized users.

## Background

Citrus are among the most popular fruit crops in the world, providing energy (carbohydrates) and nutrients, and are an important component of the daily diet in many parts of the world [35]. Fresh citrus is also a good source of dietary fibre and vitamins B and C. Citrus fruits and fruit products are globally important from nutritional and economic perspectives [56]. However, there are various biotic and abiotic challenges to citrus production, among which Huanglongbing (HLB), or citrus greening disease, is the most devastating [5, 25, 61]. HLB was first reported in China in the early 1900s and is now well established in many citrus-producing regions, including India, China, the United States, Indonesia, the Philippines, the Arabian Peninsula, Brazil, and Africa [24, 25]. Brazil and the United States produce more than 90 % of the world’s supply of orange juice, and HLB is a current threat to the U.S. and the Brazilian citrus industry. HLB was first found in 2005 in Florida, the second-largest orange producing region in the world. Since then, HLB has reached epidemic proportions in Florida and has caused more than $4 billion in economic losses between 2005 and 2011 [27]. HLB attacks all important commercial citrus, including oranges, grapefruit, and tangerines [5, 20]. Sweet oranges and mandarins are considered highly susceptible, and sour oranges and grapefruits are moderately susceptible. It seems that some lemons are tolerant to HLB and some trifoliate orange (a close relative of citrus) are resistant of HLB disease [20].

HLB is a bacterial disease caused by gram negative and phloem-restricted Liberibacter species, which are vectored by the Asian citrus psyllid (*Diaphorina citri* Kuwayama) [21, 30]. *D. citri* feeds on new leaf growth, rendering twisted and curled leaves [36]. The disease can also be transmitted to healthy trees by grafting of diseased budwood [36]. Among the three known liberibacters that cause HLB disease, *Candidatus* Liberibacter asiaticus (*Ca*Las) is the most widespread. The other two species are more geographically constrained; *Ca.* L. africanus is present primarily in Africa [30], and *Ca.* L. americanus has only been found in Brazil and China [54]. The bacterium resides in phloem tissues and causes phloem collapse, which leads to decreased productivity. The HLB disease causes a rapid tree decline with blotchy mottling of leaves, and small, misshapen, irregularly coloured, bitter fruit with aborted seeds [11, 23]. At present, there are no effective control methods for HLB, except for the use of HLB-free budwood for plant propagation, removal of infected trees to minimize inoculum, and insect vector control [5]. The full genome sequencing of *Ca*Las (1.23 Mb) has made genome-based identification of virulence factors associated with HLB symptoms possible [15]. However, *Ca*Las has not been cultured and a full understanding of the molecular basis of citrus–HLB interactions is lacking [17]. It is difficult to create HLB-tolerant citrus cultivars via conventional breeding because of a lack of any known resistance (R) genes against HLB and because of the complex biology of the citrus host. However, examination of citrus genomics through transcriptome and proteome analysis can elucidate the differentially expressed genes/proteins as potential candidate to study citrus–HLB interactions.

Several transcriptomic studies of citrus–HLB interactions have been published so far [1–3, 18, 19, 31, 34, 39–41]. These studies showed that the expression of genes in carbohydrate metabolism, nutrient transport, cell wall synthesis, and defence- and hormone-response pathways were reprogrammed by *Ca*Las infection and HLB disease development. These studies also suggested that disruption in carbohydrate source and sink pathway and phloem plugging were likely the main cause of HLB symptoms [34, 39, 40]. Numerous differentially expressed probe sets have been identified in these studies, but there was only a limited level of overlapping among the studies in terms of differentially expressed gene probe sets, possibly because of technique-, tissue-, or genotype-specific effects. Furthermore, some studies focused only on fully symptomatic plants, while others followed a time course of events during HLB development. Collective interrogation of this collection of transcriptomic studies should provide a better understanding than could be gained from analysis of individual studies. However, there are significant challenges to meta-analyses with respect to data comparability, normalization, and analysis tools. Meta-analysis of microarrays has been used extensively in animal systems to define robust, regulated probe sets [42, 45]. Recently, meta-analyses have also been used to identify differentially expressed probe sets in plants [4, 9, 50, 55]. A recent study presented a meta-analysis of HLB-responsive probe sets in citrus, but this study used a small number of datasets (six), and only one R genotype (US-897) was included for comparison [69]. To fill this gap, we performed a comprehensive meta-analysis using a larger dataset (22 studies) that included tissue-specific, susceptible, and resistance-specific HLB-responsive probe sets, and we further analysed these probe sets using co-expression and miRNA nested network approaches.

Our objective was to investigate the citrus–HLB interactions in detail using available transcriptome data and to identify robust probe sets in citrus that are regulated by *Ca*Las infection and HLB development. To do this, we analysed 46 publicly available citrus–HLB transcriptome microarray datasets from the Affymetrix GeneChip Citrus Genome Array using the Limma and RankProd methods. We identified the most statistically important tissue-specific probe sets and pathways, which we further analysed for enrichment of Gene Ontology (GO) terms using MapMan and AgriGO. In addition, we identified common probe sets that provided information on core citrus pathways and resistance-specific probe sets that gave clues about defence responses in citrus against *Ca*Las infection. Correlations among common probe sets identified with both Limma and RankProd were examined using WGCNA co-expression network analysis. We identified potential hub probe sets that showed the greatest number of interconnections and contributed most to citrus–HLB interactions. We also characterised the miRNA-mediated nested network in response to *Ca*Las infection. These findings provide potential candidate probe sets that can be used to dissect citrus–HLB interactions in detail.

## Results

### Meta-analysis of HLB-responsive transcriptome data

**Fig. 1:**
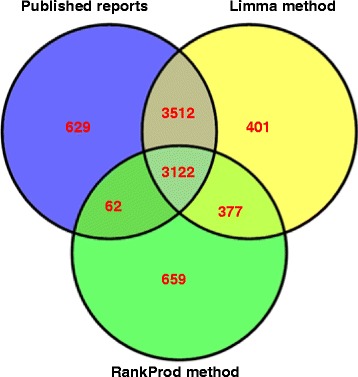
Venn diagram showing distribution of HLB-responsive differentially expressed probe sets in citrus. Figure is displaying comparison of differentially expressed probe sets of citrus-HLB interactions identified from previous published reports (blue circle) and, from the present meta-analysis by ‘Limma’ (yellow circle) and ‘RankProd’ (green circle) methods

We used 22 datasets, including 18 datasets from susceptible citrus plants (S datasets, one each for root and stem tissue, eight for leaf tissues, and eight for fruit) and four from leaf tissues of HLB-resistant citrus plants (R datasets) (Table 1). We retrieved the Gene Expression Omnibus (GEO) CEL files for the above-mentioned datasets that include 46 array samples and performed Robust MultiChip Analysis (RMA) normalisation using the affy package in the R program. Differentially expressed probe sets were identified using two statistical approaches, Limma and RankProd, in the Bioconductor package. In the Limma method, probe sets were identified based on a moderated *t* statistic, *P* < 0.05 and a false discovery rate (FDR) < 0.1. We did not use FDR < 0.05 because we could have missed many differentially expressed probe sets from some datasets (especially those for roots). The RankProd method was used under more stringent conditions (FDR < 0.01) to detect probe sets at a higher level of statistical significance. Hence, fewer probe sets were identified with RankProd than with Limma. More probe sets were identified with the Limma method also because we added common probe sets from Fan et al. [19] and fruit data [34, 40] using an in-house Teradata SQL script. We identified 7,412 probe sets using Limma and 4,221 using RankProd (Additional file 1). The above-mentioned studies reported a total of 7,326 differentially expressed probe sets. Of these, 6,634 were common in the Limma results and 3,184 were common in RankProd results (Fig. 1); 3,499 probe sets were differentially expressed in both Limma and RankProd.

**Table 1:** Details of microarray studies used for meta-analysis

Dataset	Designated in present analysis	Reference	Genotype	Number of samples	Tissue	Time after *Ca*Las infection	GSE Dataset	Analysis method	Analysis statistic	Up-regulated Probesets	Down- regulated Probesets
Root dataset	Valencia root	[3]	Valencia	6 (3C^f^ + 3I^f^)	Root	16 months	GSE33004	Limma R Package	Unadjusted *P* < 0.05 FC > 2	56	55
Stem dataset	Valencia stem	[3]	Valencia	6 (3C + 3I)	Stem	16 months	GSE33004	Limma R Package	Unadjusted *P* < 0.05 FC > 2	551	334
Susceptible (S) leaf dataset	Valencia 5–9 wai^a^	[1]	Valencia	5 (2C + 3I)	Leaf	5-9 week	GSE33459	local pooled Error (LPE)	Adjusted *P* (FDR < 0.05) FC > 2	195	84
	Valencia 13–17 wai	[1]	Valencia	5 (2C + 3I)	Leaf	13-17 week	GSE33459	local pooled Error (LPE)	Adjusted *P* (FDR < 0.05) FC > 2	310	205
	Sweet orange	[31]	sweet orange	6 (3C + 3I)	Leaf	8 months	GSE33003	Limma R Package	Unadjusted *P* < 0.05 FC > 2	307	317
	Madam Vinous	[18]	Madam Vinous	6 (3C + 3I)	Leaf	7 months	GSE29633	Limma R Package	Unadjusted *P* < 0.05 FC > 2	943	1060
	Cleopatra	[2]	Cleopatra	6 (3C + 3I)	Leaf	32 week	GSE30502	Partek suit two way ANOVA	Adjusted *P* (FDR < 0.05) FC > 4	326	68
	Madam Vinous 5 wai	[19]	Madam Vinous	6 (3C + 3I)	Leaf	5 week	Not available	Limma R Package	Unadjusted *P* < 0.05 FC > 2	83	101
	Madam Vinous 17 wai	[19]	Madam Vinous	6 (3C + 3I)	Leaf	17 week	Not available	Limma R Package	Unadjusted *P* < 0.05 FC > 2	614	414
	Madam Vinous 27 wai	[19]	Madam Vinous	6 (3C + 3I)	Leaf	27 week	Not available	Limma R Package	Unadjusted *P* < 0.05 FC > 2	1955	1523
Resistant (R) leaf dataset	RL^b^ 5 wai	[19]	Rough Lemon	6 (3C + 3I)	Leaf	5 week	Not available	Limma R Package	Unadjusted *P* < 0.05 FC > 2	239	447
	RL 17 wai	[19]	Rough Lemon	6 (3C + 3I)	Leaf	17 week	Not available	Limma R Package	Unadjusted *P* < 0.05 FC > 2	260	154
	RL 27 wai	[19]	Rough Lemon	6 (3C + 3I)	Leaf	27 week	Not available	Limma R Package	Unadjusted *P* < 0.05 FC > 2	867	190
	US-897	[2]	US-897	6 (3C + 3I)	Leaf	32 week	GSE30502	Partek suit two way ANOVA	Adjusted *P* (FDR < 0.05) FC > 4	17	none
Fruit dataset	Immature fruit	[40]	Valencia	10 (5C + 5I)	Fruit	_	SRP022979	DESeq package	_	45	24
	Mature fruit	[40]	Valencia	10 (5C + 5I)	Fruit	_	SRP022979	DESeq package	_	234	73
	Hamlin_JV^c^	[34]	Hamlin	8 (4C + 4I)	Fruit	_	GSE33373	Limma R Package	Adjusted *P* (FDR < 0.01) FC > 2	174	116
	Hamlin_VT^d^	[34]	Hamlin	8 (4C + 4I)	Fruit	_	GSE33373	Limma R Package	Adjusted *P* (FDR < 0.01) FC > 2	398	471
	Hamlin_FF^e^	[34]	Hamlin	8 (4C + 4I)	Fruit	_	GSE33373	Limma R Package	Adjusted *P* (FDR < 0.01) FC > 2	696	706
	Valencia_JV	[34]	Valencia	8 (4C + 4I)	Fruit	_	GSE33373	Limma R Package	Adjusted *P* (FDR < 0.01) FC > 2	291	110
	Valencia_VT	[34]	Valencia	8 (4C + 4I)	Fruit	_	GSE33373	Limma R Package	Adjusted *P* (FDR < 0.01) FC > 2	288	257
	Valencia_FF	[34]	Valencia	8 (4C + 4I)	Fruit	_	GSE33373	Limma R Package	Adjusted *P* (FDR < 0.01) FC > 2	210	324

^a^weeks after inoculation, ^b^Rough Lemon, ^c^Juice Vesicle, ^d^Vascular Tissue, ^e^Flavedo, ^f^Control and Infected

### Gene enrichment and pathway analysis of differentially expressed probe sets

Gene enrichment and pathway analysis of differentially expressed probe sets were conducted using MapMan and AgriGO (Additional file 2). The MapMan ontology was designed specifically for plants [59], where probe sets were assigned into largely non-redundant and hierarchically organized BINs based on their annotation. Of the 7,412 probe sets identified with Limma, 5,003 were mapped to 34 different BINs by MapMan, 2,083 fell under a ‘not assigned’ category, and the remaining 2,920 probe sets were mapped to different metabolic pathways. Signalling (*P *< 7.2e-7, cell wall (*P* < 4.9e^−4^), and carbohydrate metabolism (*P* < 0.002) were among the top significant MapMan categories. Of the 4,221 probe sets identified with RankProd, 1,703 were ‘not assigned’, and the rest 2,518 were mapped to different pathways. The RankProd analysis suggested that carbohydrate metabolism was the most significant class modulated by HLB disease in citrus.

AgriGO is a web-based tool designed specifically for GO analysis of agricultural species [14]. GO terms for differentially expressed probe sets from Limma and RankProd were assigned to biological processes, molecular functions, or cellular components. Among biological processes, ‘response to stimulus’ (Limma, FDR = 9.4e^−05^; RankProd, FDR = 0.0013) was the most significant class. Among molecular functions and cellular component terms, ‘catalytic activity’ (Limma, FDR = 0.00023; RankProd, FDR = 1.2e^−05^) and ‘extracellular region’ (Limma, FDR = 8.7e^−08^; RankProd, FDR = 9.8e^−08^) were the most significant classes. The GO analysis of differentially expressed probe sets, using hypergeometric statistics with the Yekutieli FDR multi-test adjustment (FDR < 0.01), indicated that carbohydrate metabolism, secondary metabolism, response to stimulus, cell wall metabolism, nutrient transport, and response to stress were among the most significant metabolic pathways modulated in citrus**–**HLB interactions.

To study the distribution of these six metabolic pathways (carbohydrate metabolism, cell wall metabolism, hormone metabolism, signalling, secondary metabolism, and transport) among the 22 datasets, we analysed them using the Wilcoxon test in PageMan [58]. The results were divided into an S dataset (one each from roots and stems and eight from leaves), an R dataset (resistant leaves), and a fruit dataset (Additional file 3: Figure S1, Additional file 4: Figure S2, Additional file 5: Figure S3, Additional file 6: Figure S4, Additional file 7: Figure S5, Additional file 8: Figure S6). Carbohydrate metabolism generally showed down-regulation in the fruit dataset and during early stages of *Ca*Las infection in both the R and S datasets. Probe sets related to starch degradation were up-regulated in the fruit dataset but down-regulated in the R dataset. However, starch synthesis-related probe sets were up-regulated in the resistant citrus variety US-897 and S datasets but down-regulated in the fruit dataset (Additional file 3: Figure S1). In total, 125 probe sets related to the cell wall were induced. Most cell wall-related probe sets were induced at 17 weeks after infection (wai) with *Ca*Las and were up-regulated in the R dataset and down-regulated in the S dataset. Probe sets related to cell wall synthesis, such as α-expansin 3 and pectinesterase were induced in tolerant citrus variety Rough Lemon (RL), but cell wall-modification-related probe sets were up-regulated in resistant US-897 (Additional file 4: Figure S2). In hormone metabolism, brassinosteroid and GA pathway-related probe sets were up-regulated in the R dataset. Few salicylic acid (SA)-responsive probe sets were up-regulated with ethylene-signalling probes in the S dataset (Additional file 5: Figure S3). In the signalling, all of the probe sets were up-regulated in the R dataset except RL 27 wai and coded for sugar and nutrient signalling, leucine-rich receptor, or wall-associated kinase receptor. Calcium signalling related probe sets were down-regulated in the S but not in the R dataset (Additional file 6: Figure S4). We found 217 probe sets that were related to transport. Among these, six probe sets encoding aquaporin were up-regulated only in RL 17 wai and were down-regulated in the S dataset. ABC transporters coding probe sets were up-regulated only in the S dataset (Additional file 7: Figure S5). In the defence and stress pathway, 19 probe sets coding for PR proteins or chitinase were induced in 12 datasets and showed up-regulation in R datasets (US-897 and RL 17 wai). Seventeen probe sets encoding miraculin, a member of the Kunitz serine and trypsin protease inhibitor family proteins, was also induced. Five miraculin-encoding probe sets were up-regulated in RL 17 wai, and six were down-regulated in RL 27 wai (Additional file 8: Figure S6).

### Tissue-specific gene expression in citrus–HLB interactions

**Fig. 2:**
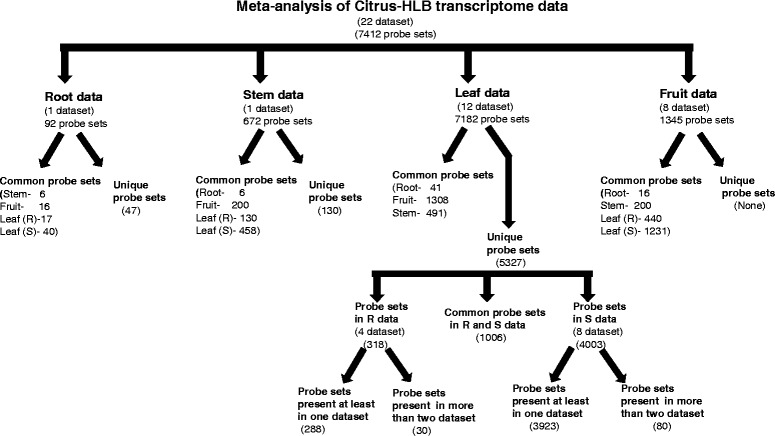
Classification of tissue-specific probe sets from 22 sample datasets. Figure represent the number of differentially expressed probe sets that are unique and common among different citrus tissues datasets (root, stem, leaf and fruit). R and S represent resistant and susceptible datasets of leaf, respectively

An in-house Teradata SQL script was used to extract fruit data corresponding to the identified 7,412 probe sets. Differentially expressed probe sets were further classified according to tissue specificity (root, stem, leaf, or fruit datasets) (Fig. 2, Additional file 9). The root dataset included 92 probe sets (Fold change >= 2 or <= -2), of which 45 were common to other datasets and the remaining 47 probe sets were specific to root data. Only 17 of these 47 probe sets were annotated in citrus or *Arabidopsis* genome databases, and they coded for Cu/Zn superoxidase dismutase, ent-kaurenoic oxidase, MYB transcription factor (two probe sets), and polygalacturonase. Stem dataset showed significant expression changes for 672 probe sets, of which 542 probe sets were common to other datasets and 130 probe sets were specific to the stem dataset. These probe sets mainly coded for transporter (*n* = 9), protein signalling (*n* = 8), and the cell wall (*n* = 6). All transport-related probe sets that coded for protein transporters (*n* = 6), peptide and oligopeptide transporters, and ABC transporters were up-regulated in HLB-diseased citrus. Cell wall-related probe sets coding for xyloglucan endotransglycosylase and expansin were down-regulated in the stem dataset. Among signalling-related probe sets, those involved in calcium signalling were down-regulated, but those related to sugar and nutrient physiology were up-regulated in stems. Most of the transcriptome studies for citrus–HLB interactions were performed using leaf tissues, and we retrieved 12 leaf datasets. A total of 7,182 probe sets showed differential expression in at least one leaf dataset, and 5,327 probe sets were unique to leaf datasets, showing no expression changes in other tissues. These 5,327 probe sets were further grouped into four R and eight S datasets; 318 probe sets were specific to the R datasets, 4,003 were specific to the S datasets, and 1,006 were common to both datasets. There were 288 probe sets present in at least one of the four R datasets, and 30 were present in more than two R datasets. From 4,003 probe sets in S datasets, 3,923 were from at least one dataset, and 80 were present in more than four datasets. For the eight fruit datasets, 1,345 probe sets showed significant fold changes in at least one dataset. Because we used fruit datasets only for comparison with our meta-analysis, no probe sets were specific to these datasets.

### Conserved expression of top common probe sets from both meta-analysis methods

**Fig. 3:**
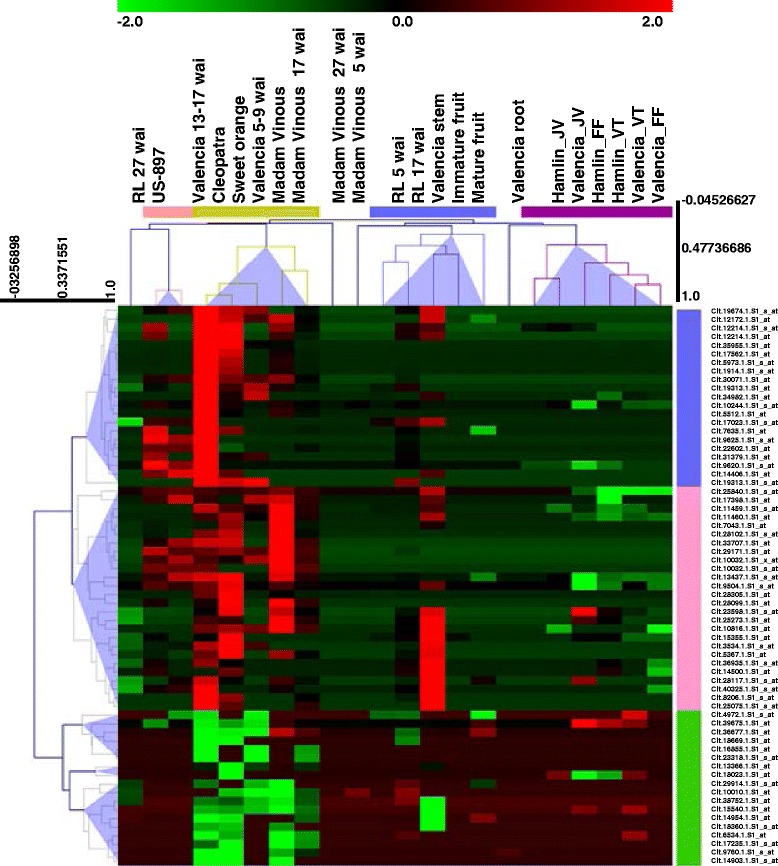
Hierarchical clustering of 65 common probe sets identified by both meta-analysis methods. The expression datasets were gene-wise normalized and, the clusters for 65 probe sets and 22 datasets were made using Pearson correlation coefficients. Heatmap showed four vertical clusters and four single nodes for 22 datasets. Five horizontal clusters were made for 65 probe sets

Using the TopGene function in RankProd, we identified the top 100 common probe sets (50 up-regulated and 50 down-regulated). We searched the status of these 100 probe sets with Limma results and taken into account only those showed differential expression at least in four datasets. Thus, the 65 most common probe sets from both methods were identified and analysed. Because these 65 probe sets are expressed in most datasets, whether or not in a tissue-specific or response-specific manner, these probe sets should signify core pathways in citrus–HLB interactions. We performed hierarchal clustering of these common probe sets with the 22 datasets using Karl Pearson correlations with average linkage [16]. The clusters revealed relatedness among the datasets for expression of the common probe sets. Using a distance threshold of 0.9, we calculated groups/nodes among differentially expressed probe sets and datasets and found five vertical clusters for probe sets and four horizontal clusters for datasets from the main hierarchical clusters (Fig. 3, Additional file 10). Cluster I comprised 21 probe sets; of these, 11 were of unknown functions, and others mainly coded for orcinol-*O*-methyltransferase (Cit.12172.1.S1_at and Cit.10244.1.S1_s_at), phloem protein 2(PP2)-B15 (Cit.35955.1.S1_at), NAC domain-containing protein (Cit.12214.1.S1_s_at), and glucose-6-phosphate/phosphate translocator 2 (GPT2) (Cit.9625.1.S1_s_at and Cit.22602.1.S1_at). Out of 21 probe sets, only four were induced in fruit datasets, and all four showed down-regulation. All probe sets were up-regulated in the R and S leaf datasets, except orcinol-*O*-methyltransferase and PP2-B15, which were not induced in any of the R datasets. Cluster II consisted of 26 probe sets that coded for Zn transporter 5 (Cit.11459.1.S1_s_at, Cit.11460.1.S1_at, and Cit.28305.1.S1_at), GASA1 (Cit.10032.1.S1_x_at), ADP-glucose pyrophosphorylase large subunit (APL3) (Cit.13437.1.S1_s_at), starch synthase (Cit.9504.1.S1_s_at), AtWRKY40 (Cit.10816.1.S1_at), CDR (Cit.28117.1.S1_s_at), Cu/Zn superoxide dismutase (Cit.28102.1.S1_s_at), and 2OG-Fe(II) oxygenase family protein (Cit.15355.1.S1_at). Cluster III consisted of six probe sets: β-amylase (Cit.39675.1.S1_at and Cit.36677.1.S1_at), nodulin (Cit.4972.1.S1_s_at), ripening-related proteins (Cit.18669.1.S1_at), and membrane protein-encoding probe sets (Cit.16855.1.S1_at). Cluster IV was made up of two probe sets that coded for chalcone synthase (Cit.13366.1.S1_at) and amino acid transport (Cit.18023.1.S1_at). Cluster V consisted of 10 probe sets mainly coding for ABC transporter (Cit.6534.1.S1_at), peroxidase (Cit.6534.1.S1_at), and 9-cis-epoxycarotenoid dioxygenase 2 (Cit.17235.1.S1_s_at). In cluster V, all of the probe sets were down-regulated in S datasets, but four were up-regulated in one R dataset (RL 17 wai).

The 22 datasets (Table 1) were grouped into four clustered and four single nodes. Madam Vinous 5 wai, Madam Vinous 27 wai, Valencia root, and RL 27 wai were not part of any cluster and remained as single nodes; US-897 and Valencia 13–17 wai were clustered together. All of the fruit data clustered together except for mature and immature fruit datasets from Martinelli et al. [40], which clustered with RL 5 wai, RL 17 wai, and the stem dataset. The remaining S datasets were clustered together. RL 5 wai and RL 17 wai showed gene responses similar to those of the stem dataset, but the US-897 response for common probe sets was similar to that for Valencia 13–17 wai.

### Differentially expressed probe sets specific to the R datasets

**Fig. 4:**
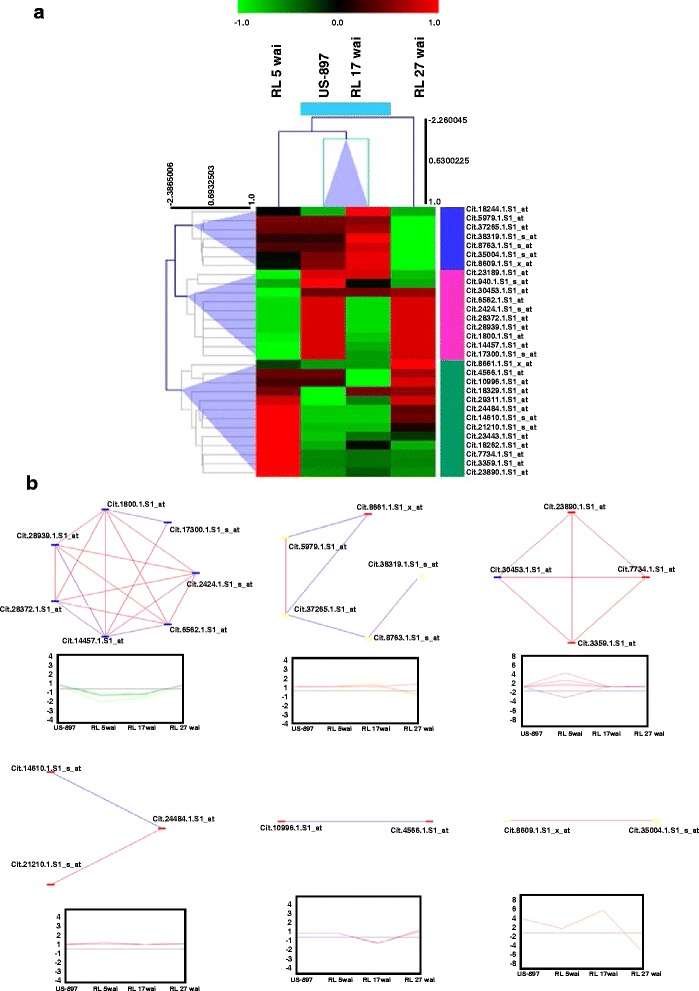
**a** Hierarchical clustering of 30 R-specific probe sets. The expression datasets were gene-wise normalized and, the clusters for 30 probe sets and four datasets were made using Pearson correlation coefficients. Heatmap showed one vertical cluster and two single nodes for four datasets. Three horizontal clusters were made for 30 probe sets. **b** Relevance network of 30 R-specific probe sets. Six relevance sub-networks were generated using 30 R-specific probe sets. Sub-networks showed that the expression of one probe set affected the expression of the other probe set either positively (shown in red line) or negatively (shown in blue line). The expression profiles of these probesets among resistant datasets (US-897, RL 5, 17 and 27 wai) are shown in rectangle boxes

Thirty differentially expressed probe sets were identified in at least two of the four R datasets (Fig. 2). The hierarchical cluster analysis using Pearson correlation at a distance threshold of 0.9 showed three vertical clusters for 30 probe sets (7, 10, and 13 probe sets) and one horizontal cluster for two R datasets (US-897 and RL 17 wai). The other two R datasets (RL 5 wai and RL 27 wai) were present as single nodes (Fig. 4a, Additional file 11). The seven probe sets comprising cluster I encoded GA-responsive protein (Cit.18244.1.S1_at), leucine-rich repeat (LRR) VIII-2 (Cit.5979.1.S1_at), terpene synthase (Cit.38319.1.S1_s_at), aquaporin (Cit.8763.1.S1_s_at), lectin-related protein precursor (Cit.35004.1.S1_s_at and Cit.8609.1.S1_x_at), and SNARE 11 (Cit.37265.1.S1_at). These seven probe sets were up-regulated in RL 17 wai and down-regulated in RL 27 wai. However, both of the lectin-related protein-coding probe sets were up-regulated in US-897 and RL 17 wai. In cluster II, eight of 10 probe sets were down-regulated in RL 5 wai and RL 17 wai encoding cinnamoyl-CoA reductase (Cit.1800.1.S1_at and Cit.28372.1.S1_at), C_2_H_2_ Zn family protein (Cit.6562.1.S1_at), DNAJ heat shock family protein (Cit.2424.1.S1_s_at), putative ABC transporter protein (Cit.23189.1.S1_at), and unknown function proteins (Cit.14457.1.S1_at, Cit.17300.1.S1_s_at, and Cit.28939.1.S1_at). One probe set from Cluster II that encoded an oxidoreductase 2OG-Fe(II) oxygenase family protein (Cit.940.1.S1_s_at) was up-regulated in US-897 and RL 17 wai. Four probe sets from Cluster III that encoded 5-AMP-activated protein kinase beta-1 subunit-related (Cit.24484.1.S1_at and Cit.14610.1.S1_s_at), abscisic stress ripening-like protein (Cit.8661.1.S1_x_at), and unknown protein (Cit.21210.1.S1_s_at) were up-regulated at RL 5 wai and RL 27 wai. Four probe sets that coded for B-box Zn family protein (Cit.18262.1.S1_at), jumonji transcription factor (Cit.7734.1.S1_at), mutT domain protein-like (Cit.23890.1.S1_at), and unknown protein (Cit.7734.1.S1_at) were up-regulated at RL 5 wai and RL 17 wai. From the remaining probe sets of Cluster III, two that encoded an unknown protein (Cit.23443.1.S1_at) were up-regulated in rough lemon at all three time points, and one that encoded NADH dehydrogenase subunit 4 (Cit.29311.1.S1_at) was down-regulated in US-897 and RL 17 wai. Cluster of US-897 and RL 17 wai suggested that both had similar expression changes against *Ca*Las infection for these 30 R-specific probe sets. However, RL 5 wai and RL 27 wai showed distinct regulation patterns of R-specific probe sets.

We further analysed the relevance network of these 30 probe sets (Fig. 4b, Additional file 11). A relevance network is a group of probe sets whose expression profiles are highly predictive of one another. Each pair of probe sets is related by a correlation coefficient greater than a minimum threshold (0.97) and less than a maximum threshold (1.0) [7]. The relevance network analysis resulted in six tightly correlated sub-networks that showed either positive or negative effects of one probe set on the expression of the other member of the pair (red and blue lines, respectively, Fig. 4b). Sub-network 1 consisted of seven probe sets that regulated one another’s gene expression in coding for unknown proteins (three probe sets), cinnamoyl CoA reductase (two probe sets), heat shock protein, and C_2_H_2_ Zn finger protein. Except for unknown protein Cit.14457.1.S1_at, which negatively regulated the expression of the other six probe sets, these probe sets positively regulated one another’s expression. Sub-network 2 consisted of five probe sets (*LRR-VIII-2*, *SNARE11*, terpene synthase, aquaporin, and abscisic stress ripening coding protein). *LRR-VIII-2* positively regulated the cell vessel transport *SNARE 11*. However, both of these probe sets were negatively regulated by abscisic stress ripening coding protein. Sub-network 3 consisted of four probe sets, two of which encoded unknown proteins, and one each for mutT domain protein-like and myo-inositol-1-phosphate synthase. Sub-network 4 included three probe sets that encoded 5-AMP-activated protein kinase beta-1 subunit-related (two probe sets) and an unknown protein. Sub-network 5 contained two probe sets, one each for diacylglycerol kinase and an unknown protein, and sub-network 6 included two probe sets for lectin-related proteins.

### Gene co-expression network analysis

**Fig. 5:**
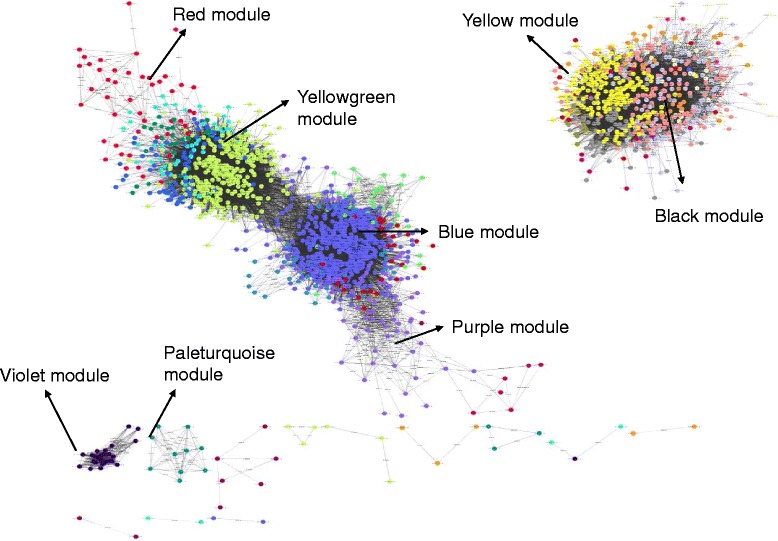
Co-expression network of 3,499 common probe sets identified in the meta-analysis. The edges with r ≥ 0.95 are shown in red and r ≤ −0.9 are show in black color. Different modules are color-coded. Circle represent a node (probe set) and edges represent connections. The network was drawn and analyzed using Cytoscape 3.1.1

Using WGCNA package [33], we constructed a weighted co-expression network of the 3,499 probe sets identified by both Limma and RankProd. This network has helped to elucidate tightly co-expressed modules and to identify hub probe sets in the respective modules [33, 46]. Our network construction yielded 2,071 nodes and 134,217 edges with a Pearson correlation coefficient of 0.85 and scale-free topological matrix. The global network was further clustered into 27 co-expressed modules using topological overlap-based average hierarchical clustering and a dissection threshold of 0.2 (Fig. 5). We computed the module eigengene (ME), which is the first principal component of each module, and we merged highly correlated MEs to yield 21 modules. A dendrogram showing gene modules before and after merging is illustrated in Additional file 12: Figure S7. A heat map was generated to visualise topological overlap values between pairs of co-expressed probe sets in the modules (Additional file 13: Figure S8). Functional analysis showed that six of the 21 modules had probe sets with overrepresented biological functions from pathways such as carbohydrate metabolism, stress, cell wall, signalling, and secondary metabolism. These six modules together contained 2,043 probe sets (Additional file 13: Figure S8: blue = 715, red = 439, black = 315, yellow = 245, magenta = 165, and purple = 164), which represented 58 % of all probe sets used for co-expressed network construction.

### Functional enrichment analysis of HLB-responsive modules and identification of hub probe sets

**Fig. 6:**
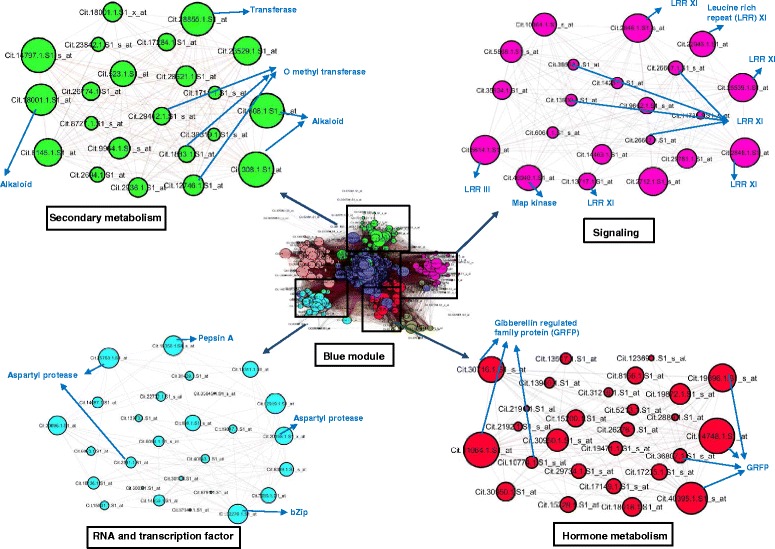
Detailed analysis of the ‘blue’ module. Figure displays secondary metabolism, signaling, RNA and transcription factor, and, hormone metabolism related sub-network with their respective hub probe sets. The edges with r ≥ 0.95 are shown in red and r ≤ −0.9 are show in black color

To understand the biological relevance of the co-expressed modules, we conducted GO and pathway analyses using AgriGO tool and MapMan. Hub probe sets having maximum connections were identified from each module (Additional file 14). Here, we describe the ‘blue’ module in detail because it contained the largest number of nodes (probe sets) and connections. Functional annotation by AgriGO revealed that carbohydrate metabolic processes (GO: 0005975, *P* = 1.7e^−10^), response to endogenous stimulus (GO: 0009719, *P* = 0.002), hydrolase activity (GO: 0016787, *P* = 6.9e^−06^), and cell wall (GO: 0005618, *P* = 9.3e^−08^) were among the most significant GO terms in the ‘blue’ module. Using MapMan analysis, nodes were classified into different metabolic pathways, including cell wall (51 nodes), RNA and transcription factors (46 nodes), signalling (45 nodes), secondary metabolism (42 nodes), hormone metabolism (33 nodes), and transport (19 nodes) (Fig. 6). Next, the top hub probe sets were identified in each category based on K_within_, and K_extracted_ >50 and node degree >500 from the 21 modules. Hub probe sets in the secondary metabolism pathway coded for alkaloids, transferase, and *O*-methyltransferase. Top probe sets in the signalling pathway encoded for LRR-XI. In relation to hormone signalling, hub probe sets encoded GA-induced proteins, and bHLH and pepsin A protease were the hub probe sets for transcription factors group. Pathway details, K_within_, and K_extracted_ for other modules are provided in Additional file 14.

We also analysed the co-expression network to identify the hub probe sets among the top 65 common and 30 R-specific probe sets. All of the top 65 common probe sets were connected among different modules in the network. For example, probe sets for lipoxygenase and *O*-methyltransferase were co-expressed in the ‘black’ module; probe sets coding for glucose transporter (GPT2) was co-expressed with *WRKY40* in the ‘grey’ module; probe sets for phloem protein (PP2-B8) and three probe sets encoding Zn transporters (ZIP5) were co-expressed in the ‘paleturquoise’ module. Probe sets for Cu/Zn superoxide dismutase, WBC11, miraculin protein, and CDR were co-expressed in the ‘midnightblue’ module (Additional file 14). Interestingly, the ‘midnightblue’ module also contained six probe sets that encoded miraculin and others that encoded disease resistance proteins such as NBS-LRR, chitinases, and LRR transmembrane protein kinase. In contrast to the top 65 common probe sets, only 11 of the 30 R-specific probe sets were co-expressed among modules in the network. Lectin protein kinase (two probe sets) and terpene synthase encoding probe sets were co-expressed in the ‘blue’ module. Three probe sets coding for nucleotide binding, Zn finger protein, and NADH dehydrogenase subunit were co-expressed in the ‘greenyellow’ module. The remaining five R-specific probe sets were distributed among different modules (Additional file 14).

### MicroRNA–probe set nested networks

**Fig. 7:**
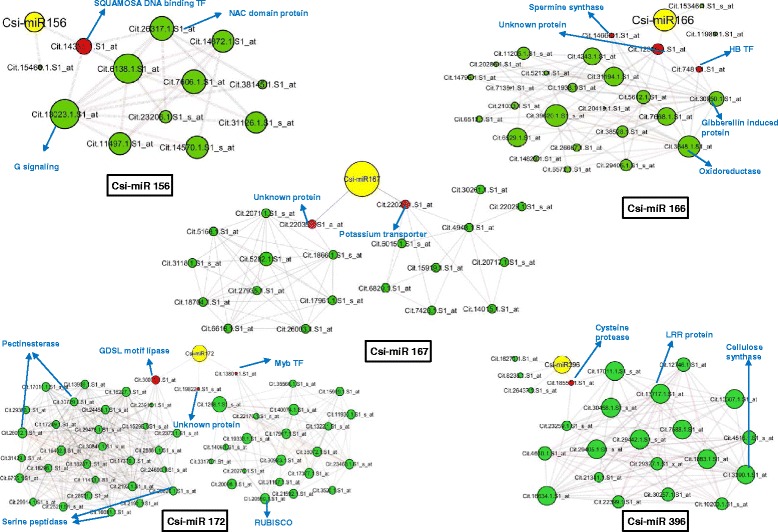
miRNA nested networks. Five miRNA families (csi-miR156, csi-miR166, csi-miR167, csi-miR172, and csi-miR396) forming nested network with their respective target probe sets. The targets are shown in red color node while miRNA classes are represented in yellow color. Connected nested networks with target probe sets are shown in green color. The edges with r ≥ 0.95 are shown in red and r ≤ −0.9 are show in black color

Recently, Zhao et al. [68] identified HLB-responsive miRNAs in susceptible citrus cultivars after 10 and 14 weeks post inoculation (wpi) with *Ca*Las. We analysed these functionally validated miRNAs and found their targets among the 3,499 common probe sets identified here. Interestingly, 10 miRNA classes were identified as targeting 24 probe sets (Additional file 15). We searched these 24 target probe sets in the co-expressed networks and used them as seed nodes to create a nested network by connecting them with first-degree neighbouring nodes. Thus, we identified nested networks for five miRNA classes (csi-miR396, csi-miR166, csi-miR156, csi-miR167, and csi-miR172) (Fig. 7).

The nested network of csi-miR396 targeted four probe sets: cytosolic ascorbate peroxidase (Cit.8235.1.S1_at), papain-like cysteine protease (Cit.26437.1.S1_s_at), nuclear matrix constituent protein (Cit.16271.1.S1_at), and cysteine protease (Cit.18551.1.S1_at). Among these, cysteine protease was linked to two large hub probe sets coding for LRR transmembrane protein kinase (Cit13717.1.S1_at) and cellulose synthase (Cit.3390.1.S1_at), which in turn were connected to the 36 stress- and secondary metabolism-related probe sets with a clustering coefficient of 0.6. The expression of csi-miR396 was reduced at both time points (10 and 14 wpi) after *Ca*Las infection [68]; therefore, its targeted probe sets should be up-regulated. Except for cytosolic ascorbate peroxidase, the other target probe sets (*n* = 3) were up-regulated in at least one S dataset (Additional file 15).

The nested network of csi-miR166 targeted five probe sets coding for unknown protein (Cit.12380.1.S1_at), HD-ZIPIII protein (Cit.7481.1.S1_at), spermine synthase (Cit.14666.1.S1_at), HD-Zip protein (Cit.15346.1.S1_s_at), and homeodomain-leucine zipper protein (Cit.11989.1.S1_at). Of these, the first three targets were connected to stress, secondary metabolism, and hormone-related hubs with 213 nodes at a clustering coefficient of 0.65. The strongest connectivity was shown by four hub probe sets coding for oxidoreductase (Cit.3648.2.S1_at), RHM1/UDP-glucose-4,6-dehydratase (Cit.39620.1.S1_s_at), ferredoxin (Cit.6529.1.S1_at), and GA-regulated protein (Cit.30950.1.S1_at). The expression of csi-miR166 was down-regulated at 14 wpi [68]. All five target probe sets were down-regulated in at least two S datasets, while unknown protein (Cit.12380.1.S1_at) was up-regulated in R datasets (RL17 wai).

The nested network of csi-miR156 targeted two probe sets, an unknown protein (Cit.14355.1.S1_at) and the SQUAMOSA promoter-binding protein (Cit.15466.1.S1_at), which were connected to the developmental pathway by three hub probe sets. These hub probe sets coded for NAC domain-containing protein (Cit.26317.1.S1_at), unknown protein (Cit.6138.1.S1_at), and G protein signalling (Cit.13023.1.S1_at). The expression of csi-miR156 was up-regulated at 14 wpi, and our data suggested that both the connected target probe sets were down-regulated in S datasets.

The nested network of csi-miR167 targeted two probe sets: an unknown protein (Cit.22035.1.S1_a_at) and potassium transporter (Cit.22029.1.S1_at). The unknown protein was connected to oxidoreductase and 2OG-Fe(II) oxygenase family protein. A potassium transporter encoding probe set was co-expressed with many transport-related probe sets, such as sulphate, phosphate (P), metal, peptide, metabolite, and ABC transporters. The expression of csi-miR167 was down-regulated at 10 wpi and up-regulated at 14 wpi in susceptible citrus [68]. Our data suggested that an unknown protein-encoding probe set was up-regulated while the potassium transport probe set was down-regulated in S datasets.

The nested network of csi-miR172 targeted three probe sets: MYB transcription factor (Cit.13801.1.S1_at), GDSL-motif lipase (Cit.30073.1.S1_at), and an unknown protein (Cit.19822.1.S1_s_at). The MYB transcription factor was connected to three hub probe sets coding for gluconeogenesis (Cit.18718.1.S1_s_at), Rubisco (Cit.20550.1.S1_at), and senescence-associated protein-related (Cit.17917.1.S1_at) at a clustering coefficient of 0.77. GDSL-motif lipase was connected with two hub probe sets coding for serine-type endopeptidase and cell wall pectinesterase family protein. Down-regulation of csi-miR172 was reported at both time points (10 and 14 wpi) in citrus [68]. Among the target probe sets, MYB transcription factor and unknown protein showed up-regulation in two S datasets, and GDSL-motif lipase showed down-regulation in one S dataset.

## Discussion

Development of HLB-resistant or tolerant citrus cultivars would be the most effective, economic, and ecologically friendly approach for managing HLB. To date, no R genes against HLB disease have been reported, and identification of the best candidate genes for functional dissection of the *Ca*Las response in citrus remains a daunting challenge. Several transcriptome studies have been conducted to identify potential citrus candidates against HLB and that have yielded more than 8,000 differentially expressed genes/probe sets (DEGs). However, information about interactions between the identified DEGs is lacking. We conducted a thorough meta-analysis and network co-expression analysis of publically available citrus transcriptome data and identified 65 common and 30 resistance-specific HLB-responsive candidate probe sets. Our study aimed to identify specific features that define the host response and targets manipulated by *Ca*Las infection and HLB development. Prior to this study, only one meta-analysis of citrus–HLB interactions was conducted [69], which included four datasets. The authors evaluated early and late HLB-responsive probe sets and suggested that hormone and defence pathways play vital roles in citrus–HLB interactions. Here, we performed a more comprehensive analysis using 22 datasets, a miRNA network, and a co-expression network to expand our understanding of citrus–HLB interactions.

We used two statistical approaches (Limma and RankProd) to identify probe sets in this meta-analysis, and we restricted the study to experiments conducted on the Affymetrix GeneChip to reduce potential technical bias in transcriptome measurements. The identified differentially expressed probe sets were subjected to Gene enrichment analysis. Our results suggested that carbohydrate metabolism, phloem plugging, transport, cell wall, hormone, defence, and stress-related pathways were the most important classes of probe sets regulated by *Ca*Las infection in citrus. We also found that very few probe sets were modulated in the citrus root dataset and those were not from any specific pathway. Further, transport-related probe sets were abundant in the stem dataset, while leaf datasets included the probe sets from many metabolic pathways. R datasets for leaves consisted mostly of cell wall-related probe sets, gibberellin signalling, and aquaporin transporters; S datasets for leaves were characterized by down-regulation of Ca signalling and up-regulation of ethylene signalling and ABC transporters. We also found that most of the HLB-responsive probe sets were modulated at intermediate stages (17 wai) rather than at early (5 wai) or late (27 wai) stages of infection. Fruit and leaf datasets were characterised by opposing directions of gene regulation; most probe sets that were up-regulated in the leaf dataset were down-regulated in fruit datasets or vice-versa. These results indicate that different tissues of a citrus plant respond in distinct ways to *Ca*Las infection and further susceptible and resistant responses of citrus add up the complexity of the interactions. To explore this further, we identified the 65 most common probe sets that were present in a maximum number of datasets and that had higher levels of expression change in their respective fold values. The 65 most common probe sets indicated that three core pathways were modulated by *Ca*Las infection. These pathways coded for carbohydrate metabolism, transporters, and hormone- and stress-related probe sets and were abundant in at least 10 of 22 citrus datasets. Hierarchical clustering of the 65 probe sets showed that six fruit datasets and five S datasets clustered separately, suggesting tight regulation of these core pathways among different tissues. Because the stem dataset was clustered with two R datasets, we speculate that stem tissues of a susceptible citrus can withstand better than its leaf tissue against *Ca*Las infection. Further, US-897 clustered with Valencia (13 and 17 wai), suggesting that 17 wai could be a crucial time point for the divergence of gene expression among susceptible and resistant citrus. We unveiled connections among common probe sets through WGCNA co-expression and miRNA nested networks. The integrated connections among these probe sets have not been characterised in earlier reports, and therefore our study suggests novel candidates for citrus–HLB interactions.

### Carbohydrate metabolism

Carbohydrate metabolism is a critical pathway in citrus–HLB interactions. HLB-diseased citrus trees are characterised by the accumulation of excessive amounts of starch and sucrose in leaves, which disrupts the starch–sucrose pathway between source (leaves) and sink (fruit) tissues. Several reports have shown that genes coding for ADP-glucose pyrophosphorylase (AGPase) large subunit, the rate-limiting enzyme in starch synthesis, was up-regulated and that β-amylase, the rate-limiting enzyme in starch degradation, was down-regulated in HLB-diseased citrus plants [1, 3, 31]. Another gene coding for glucose-6-phosphate/phosphate translocator (GPT) mediates the import of glucose-6-phosphate, an essential substrate for starch synthesis in plastids. GPT, like AGPase, is associated with the characteristic starch accumulation in HLB-affected plants [31]. In this study, these three genes were among the 65 most common probe sets. Co-expression analysis revealed that probe sets for starch synthase and AGPase were tightly connected with a hub probe set encoding NAC domain protein in the ‘plum 1’ module (Additional file 14). NAC domain transcription factors (TF) are related to plant development and have been implicated as transcriptional switches that regulate secondary wall synthesis [70]. Over-expression of NAC TFs in *Arabidopsis* leaves causes activation of secondary wall biosynthetic genes that leads to extensive deposition of secondary walls in cells [70]. Our results suggest that up-regulation of this NAC TF could control carbon deposition in citrus secondary cell walls and subsequent thickening in the leaves due to *Ca*Las infection. This study also revealed that *GPT* was co-expressed with *WRKY40*, *MYB15*, and ethylene signalling genes in the ‘grey60’ module. Previous reports also suggested the role of these genes (*WRKY40* and *MYB*) in HLB-symptomatic citrus leaves [1, 3, 31]. Interestingly, the same *MYB*-like gene was induced nearly 200-fold in symptomatic leaves of susceptible plants infected with *Ca*Las, but not in the resistant (US-897) genotype [1, 3, 31]. *MYB* genes have been reported as key regulators of sugar-responsive genes during sugar starvation in rice [37]. We suggest that the NAC domain, MYB15, and WRKY40 TFs may play important roles in the transcriptional regulation of carbohydrate metabolism in citrus–HLB interactions.

### Phloem plugging and nutrient transport

Excessive starch accumulation in citrus phloem cells induced by *Ca*Las infection affects nutrient transport and other transporter genes due to vascular blockage, caused by callose deposition and phloem plugging. Phloem protein encoding probe set (*PP2-B15*), from the 65 most common probe sets, is involved in phloem blockage [69]. Three probe sets encoding *bZIP5* were co-expressed with *PP2-B15* and ankyrin-repeat family encoding protein in the ‘paleturquoise’ module. Our findings were in agreement with Zheng and Zhao [69], who suggested that Zn transporters and phloem protein were linked in the co-expression network. *PP2-B15* was induced only in the S datasets; further, HLB symptoms resemble those caused by Zn deficiency. Hence, phloem plugging by *PP2-B15* could disrupt Zn transport in infected citrus leaves and may cause symptoms of Zn deficiency. However, the role of ankyrin-repeat protein in phloem plugging needs to be clarified. Cationic plant nutrients such as Ca, K, Mg, Fe, Cu, Mn, and Zn play important roles in citrus–HLB interactions [43]. HLB-responsive miRNA (miR399) is up-regulated during phosphorous (P) starvation in susceptible plants [68]. Our integrated miRNA nested network showed that an HLB-responsive miRNA, csi-miR167, is linked to a hub probe set that codes for potassium (K) transport, which in turn is connected to the nodes of sulphate, phosphate, metal, peptide, and metabolic transport-related probe sets. K-deficient plants tend to be more susceptible to infection than those with an adequate supply of K [49, 62]. Furthermore, it has been suggested that foliar fertilization with several mineral elements (Zn, Fe, Ca, K, and Mn) reduced HLB symptoms on infected trees [47]. Our study suggests that the expression of csi-miR167 could regulate the hub gene for K-transporters, which in turn may modulate the expression of other transport-regulated genes such as P transporters.

### Hormone and stress response

Gene reprogramming in plants against pathogen is initiated by rapid respiratory burst and redox as a part of their basal defences [48]. The 65 most common probe sets showed enrichment of oxidoreductase, 2OG-Fe(II) oxygenase, Cu/Zn superoxide dismutase, peroxidases, and catalase. Previous reports suggested that these probe sets could sequester free radicals and maintain cell homeostasis in citrus–HLB interactions [18, 43]. Present study showed that 2OG-Fe(II) oxygenase was co-expressed in the ‘Orangered4’ module connected to a hub probe set that encoded a CCCH-type Zn finger protein. Cu/Zn superoxide dismutase was co-expressed with many PR-encoding probe sets in the ‘Midnightblue’ module, which in turn was connected to a hub probe set that encoded a protease inhibitor. We suspect that up-regulation of protease inhibitor after insect vector feeding in turn may up-regulate these free radical-sequestering systems related genes in order to restrict the HLB disease-related membrane damage after *Ca*Las infection.

### R-specific probe sets in citrus–HLB interactions

We identified 30 R-specific probe sets that coded primarily for chitinase, lectins, terpene synthase, miraculins, aquaporin, GA-responsive protein, and Zn finger (C_2_H_2_ and C_2_C_2_ type) family proteins. *Ca*Las is characterised as being more parasitic than pathogenic [15] and as lacking a type III secretion system (T3SS). T3SS is required for bacterial effector proteins to be secreted into the host plant, and these effector proteins interact with host R proteins to induce a defence pathway cascade [8]. The most common group of R genes in plants is that of the nucleotide binding site-leucine-rich repeat (NBS-LRR) class. *Ca*Las is also known to suppress plant defences [61]. Here, none of the eight NBS-LRR-coding probe sets showed a specific pattern of expression in the R datasets. Similar results were reported by Aritua et al. [3], who showed that NBS-LRR and other resistance gene probe sets were down-regulated after *Ca*Las infection. The *Ca*Las genome contains most flagellin genes (*fla*), including *flg22* peptide, which could act as a pathogen-associated molecular pattern (PAMP) [71]. Flagellin, a well-characterized PAMP, is recognized by the LRR receptor kinase XII (*FLS2)* in *Arabidopsis* [32]. Here, among various LRR kinases modulated by HLB, *LRR-II* and *LRR-XII* were up-regulated in the R datasets (RL17 wai) and down-regulated in at least three S datasets. These LRR-family probe sets were also among the hub probe sets in the ‘blue’ module and were connected to a csi-miR166 target probe set encoding HD-Zip III protein. The role of these LRR probe sets in recognizing *Ca*Las effector molecules needs to be investigated.

A large number of pathogenesis-related probe sets were more abundant in the R than in the S datasets. Two of these probe sets encode1,3-β-glucanase and chitinase, and they were also implicated in *Bois noir* phytoplasma infection of grapevines [28]. Like *Ca*Las, phytoplasma (*Candidatus Phytoplasma asteris*) is a cell wall-less bacteria that parasitises plant phloem sieve cells and lacks bacterial T3SS. Co-expression network analysis showed that five probe sets coding for chitinase were co-expressed in the ‘black’ module and connected to the WRKY23-encoding hub probe set. Other R-specific probe sets coded for protease inhibitors such as miraculin and CDR. A large number of miraculin-encoding probe sets (18) were induced in citrus in response to *Ca*Las. Miraculin-like proteins have been reported in the leaves of rough lemon [57]; proteomics studies have shown higher accumulation of miraculin proteins after HLB disease [18], and miraculin-like proteins were up-regulated in lime trees by phytoplasma [53]. The present study showed that probe set coding for miraculin was co-expressed in the ‘midnightblue’ module along with PR4, MLO-like protein, and chitinase encoding probe sets. The roles of miraculins in HLB resistance require investigation. Another protease gene, *CDR*, was thought to be involved in the US-897 resistance mechanism against *Ca*Las and it encodes aspartic protease, which can release endogenous peptides for defence responses [64]. Our results showed that *Ca*Las-induced csi-miR396 targets protease probe sets that are co-expressed with LRR receptor hubs, indicating a role of proteases and LRR receptor kinase in HLB defence responses. It should be noted that expression of csi-miR396 decreased with *Ca*Las infection. Reduced expression of miR396 was also documented in transgenic *Arabidopsis* expressing phytoplasma effector protein SAP11 [38]. It has been suggested that miR396 is required for cell proliferation during root and leaf development [38]. The role of miR396 in HLB defence mechanisms needs to be clarified in future studies.

Citrus defence against HLB disease seems not to be characterised by jasmonic acid (JA) and/or salicylic acid (SA) signalling [31, 61]. *Ca*Las encodes salicylate hydroxylase, which converts SA into catechol, a product that does not induce resistance [60]. The effects of different plant growth regulators have been studied in phytoplasma-affected plants [10]. Phytoplasma are also known to suppresses JA signalling responses in plant against insect vectors and also down-regulate the SA-mediated defence responses against bacterial effector protein SAP11 [38]. Phytoplasma manipulates development and defence hormone biosynthesis by suppressing the JA pathway and by modulating phosphate homeostasis through triggering the P starvation response [52]. Down-regulation of the JA pathway and P starvation has also been documented in *Ca*Las infection [31, 68]. Zhao et al. [68] reported that phosphate deficiency worsened HLB symptoms, while application of exogenous P reduced the symptoms. It has also been suggested that under P starvation, plants accumulate sugars and starch in their leaves [26]. In the present study, LRR receptor kinase and PR encoding probe sets were co-expressed with the GA signalling pathway related probe sets. GA signalling-related genes such as *GASA* and *GAST* were up-regulated in the R datasets but down-regulated in the S datasets. Our results were in concordance with Martinelli et al. [41] showing an opposite pattern of GA-related genes expression; down regulated in fruits but up regulated in leaves. The authors [41] also suspected that change in fruit sugar metabolism might be linked with the down-regulation of GA pathway which in turn regulate energy and carbohydrate metabolism. It has also been documented that *GASA5* was constitutively more expressed in resistant US-897, and expression levels were significantly induced in response to infection [2]. GA deficiency has been implicated in phytoplasma diseases [12]. Ding et al. [12] suggested that in potato purple-top phytoplasma infection in tomato, increased GA signalling is required to activate the defence-related enzymes β-1,3-glucanase and chitinase. We propose that GA signalling could co-ordinate defence responses in citrus–HLB interactions. Effect of different plant growth regulators on phytoplasma affected plants has been studied [10].

We found that lectin precursor probe sets were up-regulated in US-897 and RL 17 wai but not in the S datasets. Lectins are a unique group of plant proteins that can recognise and bind to glycol conjugates present on the external bacterial surface [44]. Weintraub [63] reported that lectins might have direct effects on the insect vector during phytoplasma infection. Snowdrop lectins bind to the insect midgut and are not fully degraded by midgut proteases. Because *Ca*Las is mediated through the insect vector citrus psyllid, we propose that lectins may have direct roles by inhibiting insect feeding on citrus plants. Engineering plants with these lectin genes may deter citrus psyllid, and subsequently HLB incidence.

## Conclusion

Here, we have assembled currently available transcriptome data on differential gene expression in citrus after *Ca*Las infection and upgraded it from mere a ‘list’ to ‘interconnections’ through co-expression and miRNA network analyses. By integrating transcriptomic information, we identified major hub genes for susceptible and resistant response of citrus–HLB interaction. Our data showed that carbohydrate metabolism is regulated by hub genes coding for MYB, NAC and WRKY transcription factor. Further reduced ion transport in susceptible plants could be restore by regulating csi-miR167. Our analysis identified 30 R-specific probe sets as potential candidates against HLB disease. We hypothesise that basal defences in citrus against HLB could be mediated by degradation of *Ca*Las -derived PAMPs such as flagellin or by other unknown effectors which could be recognised by LRR family receptor proteins and chitinases. R gene-mediated defences against HLB may be mediated by plant proteases such as miraculin and CDR. Analysis also suggest that citrus proteases are regulated by csi-miR396 after *Ca*Las infection and csi-miR396 may have a role in defence pathway. Our study indicate that GA signalling could play a vital role in HLB resistance.

## Methods

### Microarray data acquisition and pre-processing

We included 14 studies from six reports that are available from the NCBI Gene Expression Omnibus (GEO) database (http://www.ncbi.nlm.nih.gov/geo) [1–3, 18, 19, 31]. All of these studies were based on the Affymetrix system. The GEO series datasets were available for eight studies from five reports (GenBank: GSE33004, GSE33003, GSE33459, GSE30502, and GSE29633). The remaining six studies were from the data of Fan et al. [19]. The 14 studies included one dataset for roots (GenBank: GSE33004), one dataset for stems (GenBank: GSE33004), and 12 datasets for leaf tissues (GenBank: GSE29633, GSE30502, GSE33003, GSE33459, and Fan et al. [19]). Our study did not include meta-analysis of fruit tissues, but we included fruit transcriptome data from two published studies [34, 40] and compared it with our dataset using an in-house Teradata database SQL script (http://www.teradata.com/products-and-services/database). From different combinations of fruit studies mentioned in these reports, we included HLB-symptomatic fruit probe sets against their respective healthy controls in order to reduce the complexity of the fruit data. We included six fruit datasets from Liao and Burns [34] and two datasets from Martinelli et al. [40]. Therefore, this study analyse transcriptional changes in HLB-responsive probe sets from 22 datasets after including eight studies on fruit datasets. These 22 datasets include different time points (early and late time points after *Ca*Las infection), different tissues (roots, stems, leaves, and fruit), and different host responses against HLB disease (susceptibility and tolerance/resistance). The susceptible group consisted of 18 datasets, and the resistant group included four datasets. The accession numbers for the data sets are indicated in Table 1. Background correction and normalisation of the raw data sets were performed using RMA implemented in the affy package in R [22].

### Statistical analysis and identification of differentially expressed probe sets

To identify differentially expressed probe sets, two meta-analysis approaches were applied to the normalized gene expression datasets. The first approach, the Limma method (linear modelling of microarray data) used the Limma package of Bioconductor [51]. Limma computes moderated *t*-statistics and log-odds of differential expression by empirical Bayes shrinkage of standard errors towards a common value. Probe sets with *P* < 0.05 (FDR < 0.1) and fold change were considered. Limma generated separate files for each study, and these files were combined using an in-house SQL script on the Teradata database (http://www.teradata.com/products-and-services/database). A second nonparametric meta-analysis, the RankProd method, combines *P* values for identifying probe sets from individual experiments [6]. We used the RPadvance function in the Bioconductor package [29], which is specifically designed for meta-analysis. The RankProd output was a single file in the form of Tables 1 and 2 (down-regulated and up-regulated HLB-responsive probe sets, respectively), gene expression, FC, *P*-values, and percentage of false predictions (PFP). The number of permutation tests was set to 250, and the TopGene function with a PFP cut-off value of <0.01 was used to identify the top differentially expressed probe sets among the studies.

### Functional enrichment analysis of differentially expressed probe sets

We used the singular enrichment analysis method AgriGO (http://bioinfo.cau.edu.cn/agriGO/) with default settings for Fisher’s *t*-test (*P* < 0.05) and FDR correction by the Hochberg method for the GO analysis. Probe sets were annotated against the *Arabidopsis* genome, and citrus orthologs were identified using the HarvEST database (http://harvest-web.org). Functions and metabolic pathways of probe sets were visualized using MapMan with the *Citrus sinensis* mapping file (Csinensis_154.txt) (http://mapman.gabipd.org/). The PageMan analysis plugin of MapMan was used to visualise differences among HLB-responsive tissue-specific metabolic pathways using Wilcoxon tests, no correction, and an over-representation analysis (ORA) cutoff value of 1.0. Hierarchical clustering of microarray data, using Pearson correlation and average linkage method with distance threshold of 0.90, was performed using multiarray viewer software from TIAR (http://www.tm4.org/mev.html).

### Weighted gene co-expression network construction

Differentially expressed probe sets were taken to infer a weighted gene co-expression network by using the WGCNA package in R version 1.27.1 [33]. Pairwise Pearson correlations (*r*^2^) were calculated for probe sets across all samples to find correlations and to generate similarity matrices. Similarity (S_*ij*_) in correlation between the *i*^th^ and *j*^th^ probe sets was calculated using the following equation [6]: S_*ij*_ = abs|cor(X_*i*_ – X_*j*_)|, where, X_*i*_ and X_*j*_ = log (base 2) ratio of expression of the *i*th and *j*th genes across the samples, respectively; cor = Pearson correlation coefficient; and abs = absolute value. The absolute value of the Pearson correlation coefficient was used to generate an undirected, weighted network. The similarity matrix was weighted to adjacency matrices by raising it to a power (β). The PickSoftThreshold function of WGCNA was used to choose the appropriate power for the network topology from various soft-thresholding powers. The scale-free network was rendered by raising the soft thresholding power (β) to 14. At this threshold power, the model was fitted with *r*^2^ < 0.85. This similarity matrix was transformed into the adjacency matrix, which was transformed into a topological overlap matrix (TOM) similarity measure, a robust measure of pairwise interconnectedness [65]. The TOM matrix of probe sets was coupled with average linkage hierarchical clustering to cluster the probe sets into distinct modules using the Dynamic Tree Cut algorithm (cutreeDynamic method; deepSplit = 3, cutheight = 0.993; minimal module size = 40) [33]. Further, similar modules were merged using parameter cut height = 0.2. The co-expressed network was visualised using Cytoscape version 3.1.1 and analysed using the Network Analyser plugin [13]. We considered edges only above a threshold of 0.2 to simplify and concentrate on relevant functions of the co-expressed network. Hub probe sets in the constructed network were identified as suggested by Puniya et al. [46].

### miRNA target prediction and nested network construction

We obtained the list of miRNAs that targeted HLB-responsive probe sets from three sources: PMRD, the plant miRNA database [67], and the *Citrus sinensis* annotation project (http://citrus.hzau.edu.cn/orange/), and validated HLB-responsive miRNAs from Zhao et al. [68]. Target genes interacting with each HLB responsive miRNA class from Zhao et al. [68] were searched in the *Citrus sinensis* annotation project (http://citrus.hzau.edu.cn/orange/). We identified 64 target genes for 14 miRNA classes. The corresponding probe sets for these 64 target genes were searched from our 7,412 differentially expressed probe sets based on common *Arabidopsis* annotation and NCBI BLASTX (http://blast.ncbi.nlm.nih.gov/), resulting 24 target probe sets for 10 miRNA classes. The nested networks of 10 miRNAs and their 24 target probe sets were inferred using the nested network plugin in Cytoscape 3.1.1 with default settings.

## Additional files

Additional file 1:
**Probe sets identified in the present analysis.** List of the total number of probe sets identified with Limma and RankProd methods of meta-analysis. Additional file 2:
**Gene Ontology of identified probe sets.** Gene enrichment analysis based on MapMan and AgriGO. Additional file 3: Figure S1. PageMan display of coordinated changes of “major CHO metabolism” gene categories in 22 data set. Additional file 4: Figure S2. PageMan display of coordinated changes of “cell wall” gene categories in 22 data set. Additional file 5: Figure S3. PageMan display of coordinated changes of “hormone metabolism” gene categories in 22 data set. Additional file 6: Figure S4. PageMan display of coordinated changes of “signaling” gene categories in 22 data set. Additional file 7: Figure S5. PageMan display of coordinated changes of “transport” gene categories in 22 data set. Additional file 8: Figure S6. PageMan display of coordinated changes of “stress” gene categories in 22 data set. Additional file 9:
**List of tissue-specific probe sets.** Description of unique and common tissue-specific probe sets identified in root, stem, leaf and fruit datasets. Additional file 10:
**List of 65 top common probe sets and their expression values.** Description of hierarchical clusters identified in the top 65 common probe sets and their fold values among 22 datasets. Additional file 11:
**List of 30 R-specific probe sets and their expression values.** Description of hierarchical clusters identified in 30 R-specific probe sets and their fold values among four HLB-resistant datasets (Sheet 1). List of probe sets present in relevance network (Sheet 2). Additional file 12: Figure S7. Dendrogram for co-expressed modules. Dendrogram showing co-expressed modules before and after merging using the WGCNA package. Additional file 13: Figure S8. Network Heatmap plot for co-expressed modules. Heatmap view of topological overlap values in different modules. Red color shows highly co-expressed probe sets forming a module. Additional file 14:
**Description of WGCNA networks and hub genes.** Co-expression module connectivity and description for the identified 21 modules. Description of common and R-specific probe sets among different co-expression modules. Additional file 15:
**Detail of miRNA- probe set interactions.** Description of 10 miRNA classes and their 24 targeted probe sets with respective expression profile among 22 datasets.

## Abbreviations

ACP: Asian citrus psyllid
AGPase: ADP-glucose pyrophosphorylase
*Ca*Las: *Candidatus* Liberibacter asiaticus
CDR: Constitutive disease resistance
FDR: False discovery rate
GA: Gibberellin
GEO: Gene expression omnibus
GO: Gene ontology
GPT: Glucose-6-phosphate/phosphate translocator
HLB: Huanglongbing
JA: Jasmonic acid
ME: Module eigengene
NBS-LRR: Nucleotide-binding site leucine-rich repeat
P: Phosphorous
*P*: Probability value
PP2: Phloem protein
PR: Pathogenesis-related
R: Resistant
RL: Rough lemon
RMA: Robust multiChip analysis
S: Susceptible
SA: Salicylic acid
SQL: Structured query language
WGCNA: Weighted gene co-expression network analysis

## References

[1] Albrecht U, Bowman KD (2008). Gene expression in *Citrus sinensis* (L.) Osbeck following infection with the bacterial pathogen ‘*Candidatus Liberibacter asiaticus’* causing Huanglongbing in Florida. Plant Sci.

[2] Albrecht U, Bowman KD (2012). Transcriptional response of susceptible and tolerant citrus to infection with ‘*Candidatus Liberibacter asiaticus’*. Plant Sci.

[3] Aritua V, Achor D, Gmitter FG, Albrigo G, Wang N (2013). Transcriptional and microscopic analyses of citrus stem and root responses to ‘*Candidatus Liberibacter asiaticus’* infection. PloS One.

[4] Bhargava A, Clabaugh I, To JP, Maxwell BB, Chiang YH, Schaller GE (2013). Identification of cytokinin-responsive genes using microarray meta-analysis and RNA-seq in *Arabidopsis*. Plant Physiol.

[5] Bové JM (2006). Huanglongbing: a destructive, newly-emerging, century-old disease of citrus. J Plant Pathol.

[6] Breitling R, Amtmann A, Herzyk P (2004). Graph-based iterative group analysis enhances microarray interpretation. BMC Bioinformatics.

[7] Butte AJ, Tamayo P, Slonim D, Golub TR, Kohane IS (2000). Discovering functional relationships between RNA expression and chemotherapeutic susceptibility using relevance networks. Proc Natl Acad Sci USA.

[8] Buttner D, He SY (2009). Type III protein secretion in plant pathogenic bacteria. Plant Physiol.

[9] Canales J, Moyano TC, Villarroel E, Gutiérrez RA (2014). Systems analysis of transcriptome data provides new hypotheses about *Arabidopsis* root response to nitrate treatments. Front Plant Sci.

[10] Chang C (1998). Pathogenicity of aster yellows phytoplasma and *Spiroplasma citri* on periwinkle. Phytopathology.

[11] Da Graca J, Korsten L, Naqvi S (2004). Citrus Huanglongbing: Review, present status and future strategies. Diseases of Fruits and Vegetables.

[12] Ding Y, Wu W, Wei W, Davis R, Lee I, Hammond R (2013). Potato purple top phytoplasma‐induced disruption of gibberellin homeostasis in tomato plants. Ann Appl Biol.

[13] Doncheva NT, Assenov Y, Domingues FS, Albrecht M (2012). Topological analysis and interactive visualization of biological networks and protein structures. Nat Protoc.

[14] Du Z, Zhou X, Ling Y, Zhang Z, Su Z (2010). AgriGO: a GO analysis toolkit for the agricultural community. Nucleic Acids Res.

[15] Duan Y, Zhou L, Hall DG, Li W, Doddapaneni H, Lin H (2009). Complete genome sequence of citrus Huanglongbing bacterium, *‘Candidatus Liberibacter asiaticus’* obtained through metagenomics. Mol Plant-Microbe Interact.

[16] Eisen MB, Spellman PT, Brown PO, Botstein D (1998). Cluster analysis and display of genome-wide expression patterns. Proc Natl Acad Sci USA.

[17] Fagen JR, Leonard MT, McCullough CM, Edirisinghe JN, Henry CS, Davis MJ (2014). Comparative genomics of cultured and uncultured strains suggests genes essential for free-living growth of Liberibacter. PloS One.

[18] Fan J, Chen C, Yu Q, Brlansky RH, Li Z, Gmitter FG (2011). Comparative iTRAQ proteome and transcriptome analyses of sweet orange infected by *‘Candidatus Liberibacter asiaticus’*. Physiol Plantarum.

[19] Fan J, Chen C, Yu Q, Khalaf A, Achor DS, Brlansky RH (2012). Comparative transcriptional and anatomical analyses of tolerant rough lemon and susceptible sweet orange in response to *‘Candidatus Liberibacter asiaticus’* Infection. Mol Plant-Microbe Interact.

[20] Folimonova SY, Robertson CJ, Garnsey SM, Gowda S, Dawson WO (2009). Examination of the responses of different genotypes of citrus to Huanglongbing (citrus greening) under different conditions. Phytopathology.

[21] Garnier M, Danel N, Bove´ J. The greening organism is a gram negative bacterium. In: Garnsey SM, Timmer LW, Dodds JA, editors. Proceedings of the Ninth Conference of the International Organization of Citrus Virologists. Riverside CA; 1984. p. 115–124.

[22] Gautier L, Cope L, Bolstad BM, Irizarry RA (2004). affy--analysis of Affymetrix GeneChip data at the probe level. Bioinformatics.

[23] Gottwald TR (2010). Current epidemiological understanding of citrus Huanglongbing. Annu Rev Phytopathol.

[24] Gottwald T, Bassanezi R, Amorim L, Bergamin-Filho A (2007). Spatial pattern analysis of citrus canker-infected plantings in São Paulo, Brazil, and augmentation of infection elicited by the Asian leafminer. Phytopathology.

[25] Halbert SE, Manjunath KL (2004). Asian citrus psyllids (*Sternorrhyncha: Psyllidae*) and greening disease of citrus: a literature review and assessment of risk in Florida. Fla Entomol.

[26] Hammond JP, White PJ (2011). Sugar signaling in root responses to low phosphorus availability. Plant Physiol.

[27] Hodges AW, Spreen TH (2012). Economic impacts of citrus greening (HLB) in Florida, 2006/07–2010/11.

[28] Holton N, Nekrasov V, Ronald PC, Zipfel C (2015). The phylogenetically-related pattern recognition receptors EFR and XA21 recruit similar immune signaling components in monocots and dicots. PLoS Pathog.

[29] Hong F, Breitling R, McEntee CW, Wittner BS, Nemhauser JL, Chory J (2006). RankProd: a bioconductor package for detecting differentially expressed genes in meta-analysis. Bioinformatics.

[30] Jagoueix S, Bove JM, Garnier M (1994). The phloem-limited bacterium of greening disease of citrus is a member of the alpha subdivision of the Proteobacteria. Int J Syst Bacteriol.

[31] Kim J, Sagaram US, Burns JK, Li J, Wang N (2009). Response of sweet orange (*Citrus sinensis*) to *‘Candidatus Liberibacter asiaticus’* infection: microscopy and microarray analyses. Phytopathology.

[32] Landi L, Romanazzi G (2011). Seasonal variation of defense-related gene expression in leaves from Bois noir affected and recovered grapevines. J Agric Food Chem.

[33] Langfelder P, Horvath S (2008). WGCNA: an R package for weighted correlation network analysis. BMC Bioinformatics.

[34] Liao HL, Burns JK (2012). Gene expression in *Citrus sinensis* fruit tissues harvested from Huanglongbing-infected trees: comparison with girdled fruit. J Exp Bot.

[35] Liu Y, Heying E, Tanumihardjo SA (2012). History, global distribution, and nutritional importance of citrus fruits. Compr Rev Food Sci F.

[36] Lopes S, Bertolini E, Frare G, Martins E, Wulff N, Teixeira D (2009). Graft transmission efficiencies and multiplication of *‘Candidatus Liberibacter americanus’* and *‘Ca. Liberibacter asiaticus’* in citrus plants. Phytopathology.

[37] Lu CA, Ho TH, Ho SL, Yu SM (2002). Three novel MYB proteins with one DNA binding repeat mediate sugar and hormone regulation of alpha-amylase gene expression. Plant Cell.

[38] Lu YT, Li MY, Cheng KT, Tan CM, Su LW, Lin WY (2014). Transgenic plants that express the phytoplasma effector SAP11 show altered phosphate starvation and defense responses. Plant Physiol.

[39] Mafra V, Martins PK, Francisco CS, Ribeiro-Alves M, Freitas-Astua J, Machado MA (2013). *Candidatus Liberibacter americanus’* induces significant reprogramming of the transcriptome of the susceptible citrus genotype. BMC Genomics.

[40] Martinelli F, Reagan RL, Uratsu SL, Phu ML, Albrecht U, Zhao W (2013). Gene regulatory networks elucidating Huanglongbing disease mechanisms. PloS One.

[41] Martinelli F, Uratsu SL, Albrecht U, Reagan RL, Phu ML, Britton M (2012). Transcriptome profiling of citrus fruit response to Huanglongbing disease. PloS One.

[42] Nazri A, Lio P (2012). Investigating meta-approaches for reconstructing gene networks in a mammalian cellular context. PloS One.

[43] Nwugo CC, Duan Y, Lin H (2013). Study on citrus response to Huanglongbing highlights a down-regulation of defense-related proteins in lemon plants upon ‘*Ca. Liberibacter asiaticus’* infection. PloS One.

[44] Peumans WJ, Van Damme EJ (1995). Lectins as plant defense proteins. Plant Physiol.

[45] Plank J, Siebenhofer A, Berghold A, Jeitler K, Horvath K, Mrak P (2005). Systematic review and meta-analysis of short-acting insulin analogues in patients with diabetes mellitus. Arch Intern Med.

[46] Puniya BL, Kulshreshtha D, Verma SP, Kumar S, Ramachandran S (2013). Integrated gene co-expression network analysis in the growth phase of *Mycobacterium tuberculosis* reveals new potential drug targets. Mol BioSyst.

[47] Pustika A, Subandiyah S, Holford P, Beattie G, Iwanami T, Masaoka Y (2008). Interactions between plant nutrition and symptom expression in mandarin trees infected with the disease Huanglongbing. Australas Plant Dis Notes.

[48] Rawat N, Neeraja CN, Nair S, Bentur JS (2012). Differential gene expression in gall midge- susceptible rice genotypes revealed by suppressive subtraction hybridization (SSH) cDNA libraries and microarray analysis. Rice.

[49] Sarwar M (2012). Management of rice stem borers (*Lepidoptera: Pyralidae*) through host plant resistance in early, medium and late plantings of rice (*Oryza sativa* L.). J Cereals Oilseeds.

[50] Shaik R, Ramakrishna W (2014). Machine learning approaches distinguish multiple stress conditions using stress-responsive genes and identify candidate genes for broad resistance in rice. Plant Physiol.

[51] Smyth GK (2005). Limma: linear models for microarray data. Bioinformatics and computational biology solutions using R and Bioconductor.

[52] Sugio A, MacLean AM, Hogenhout SA (2014). The small phytoplasma virulence effector SAP11 contains distinct domains required for nuclear targeting and CIN‐TCP binding and destabilization. New Phytol.

[53] Taheri F, Nematzadeh G, Zamharir MG, Nekouei MK, Naghavi M, Mardi M (2011). Proteomic analysis of the Mexican lime tree response to *‘Candidatus Phytoplasma aurantifolia’* infection. Mol BioSyst.

[54] Texeira DC, Ayres J, Kitajima E, Danet L, Jagoueix-Eveillard S, Saillard C, et al. First report of a Huanglongbing-like disease of citrus in Sao Paulo State, Brazil and association of a new Liberibacter species, *‘Candidatus Liberibacter americanus’*, with the disease. Plant Dis. 2005;89(1):107–7. 10.1094/PD-89-0107A 30795297

[55] Thirunavukkarasu N, Hossain F, Mohan S, Shiriga K, Mittal S, Sharma R (2013). Genome-wide expression of transcriptomes and their co-expression pattern in subtropical maize (*Zea mays* L.) under waterlogging stress. PloS One.

[56] Ting SV, Nagy S, Attaway JA (1980). Nutrients and nutrition of citrus fruits. Citrus Nutrition and Quality.

[57] Tsukuda S, Gomi K, Yamamoto H, Akimitsu K (2006). Characterization of cDNAs encoding two distinct miraculin-like proteins and stress-related modulation of the corresponding mRNAs in *Citrus jambhiri* Lush. Plant Mol Biol.

[58] Usadel B, Nagel A, Steinhauser D, Gibon Y, Blasing OE, Redestig H (2006). PageMan: an interactive ontology tool to generate, display, and annotate overview graphs for profiling experiments. BMC Bioinformatics.

[59] Usadel B, Nagel A, Thimm O, Redestig H, Blaesing OE, Palacios-Rojas N (2005). Extension of the visualization tool MapMan to allow statistical analysis of arrays, display of corresponding genes, and comparison with known responses. Plant Physiol.

[60] Van Loon L, Bakker P, Pieterse C (1998). Systemic resistance induced by rhizosphere bacteria. Annu Rev Phytopathol.

[61] Wang N, Trivedi P (2013). Citrus Huanglongbing: a newly relevant disease presents unprecedented challenges. Phytopathology.

[62] Wang M, Zheng Q, Shen Q, Guo S (2013). The critical role of potassium in plant stress response. Int J Mol Sci.

[63] Weintraub PG (2007). Insect vectors of phytoplasmas and their control-an update. B Insectol.

[64] Xia Y, Suzuki H, Borevitz J, Blount J, Guo Z, Patel K (2004). An extracellular aspartic protease functions in *Arabidopsis* disease resistance signaling. EMBO J.

[65] Yip AM, Horvath S (2007). Gene network interconnectedness and the generalized topological overlap measure. BMC Bioinformatics.

[66] Zhang B, Horvath S (2005). A general framework for weighted gene co-expression network analysis. Statistical applications in genetics and molecular biology.

[67] Zhang Z, Yu J, Li D, Zhang Z, Liu F, Zhou X (2010). PMRD: plant microRNA database. Nucleic Acids Res.

[68] Zhao H, Sun R, Albrecht U, Padmanabhan C, Wang A, Coffey MD (2013). Small RNA profiling reveals phosphorus deficiency as a contributing factor in symptom expression for citrus Huanglongbing disease. Mol Plant.

[69] Zheng ZL, Zhao Y (2013). Transcriptome comparison and gene coexpression network analysis provide a systems view of citrus response to *‘Candidatus Liberibacter asiaticus’* infection. BMC Genomics.

[70] Zhong R, Demura T, Ye ZH (2006). SND1, a NAC domain transcription factor, is a key regulator of secondary wall synthesis in fibers of *Arabidopsis*. Plant Cell.

[71] Zou H, Gowda S, Zhou L, Hajeri S, Chen G, Duan Y (2012). The destructive citrus pathogen, ‘*Candidatus Liberibacter asiaticus’* encodes a functional flagellin characteristic of a pathogen-associated molecular pattern. PloS One.

