# Supplementary figures and images for: Comprehensive meta-analysis, co-expression, and miRNA nested network analysis identifies gene candidates in citrus against Huanglongbing disease
Source: BMC Plant Biol. 2015 Jul 28;15:184. doi: 10.1186/s12870-015-0568-4 (PMC4517500; doi:10.1186/s12870-015-0568-4)

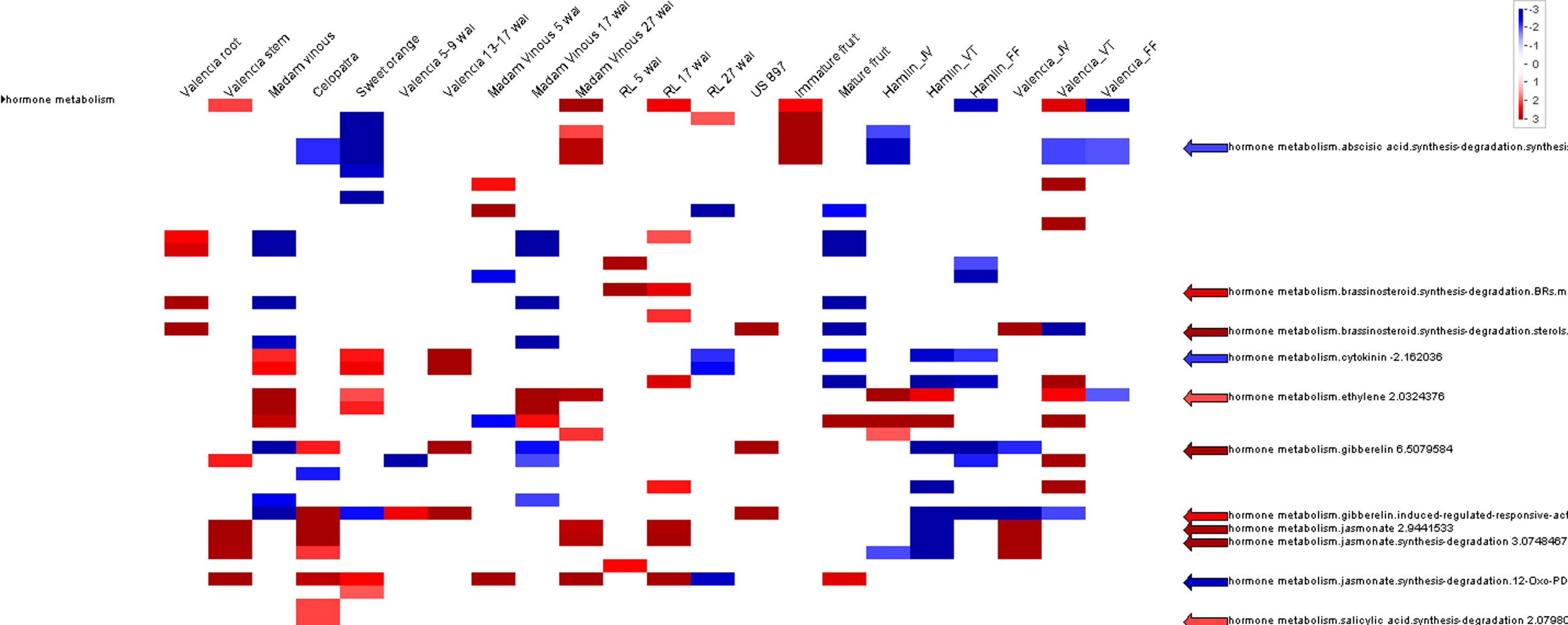

Supplement: Additional file 5: Figure S3. — PageMan display of coordinated changes of “hormone metabolism” gene categories in 22 data set. [file 12870_2015_568_MOESM5_ESM.jpg]

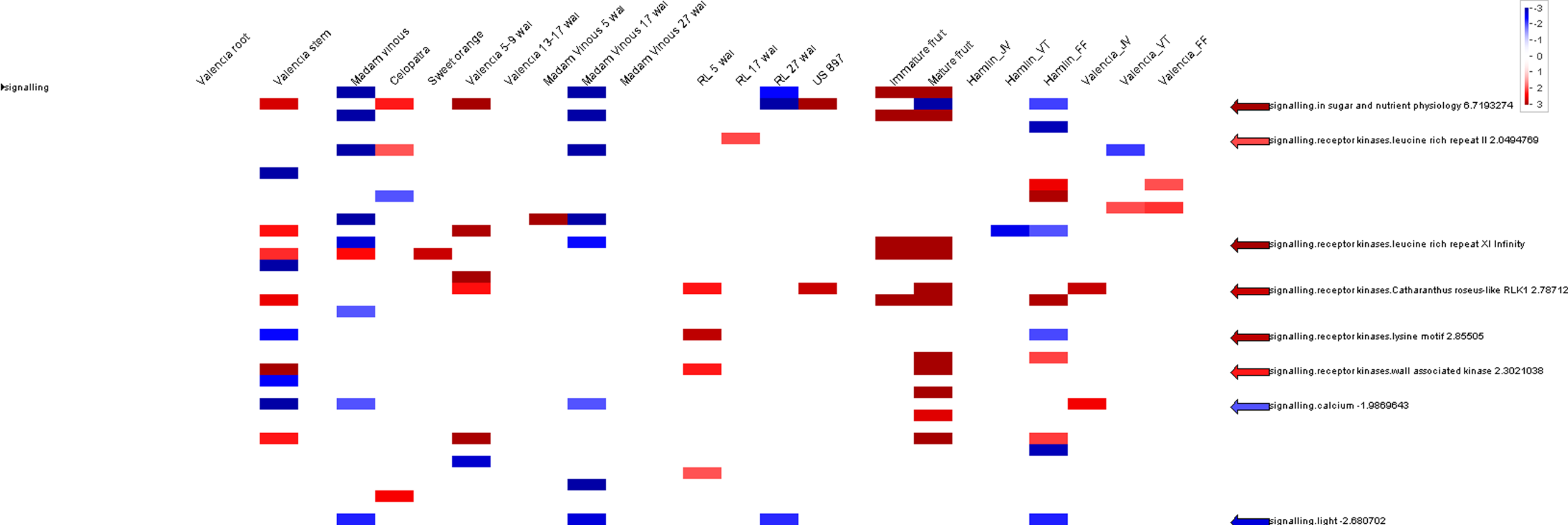

Supplement: Additional file 6: Figure S4. — PageMan display of coordinated changes of “signaling” gene categories in 22 data set. [file 12870_2015_568_MOESM6_ESM.jpg]

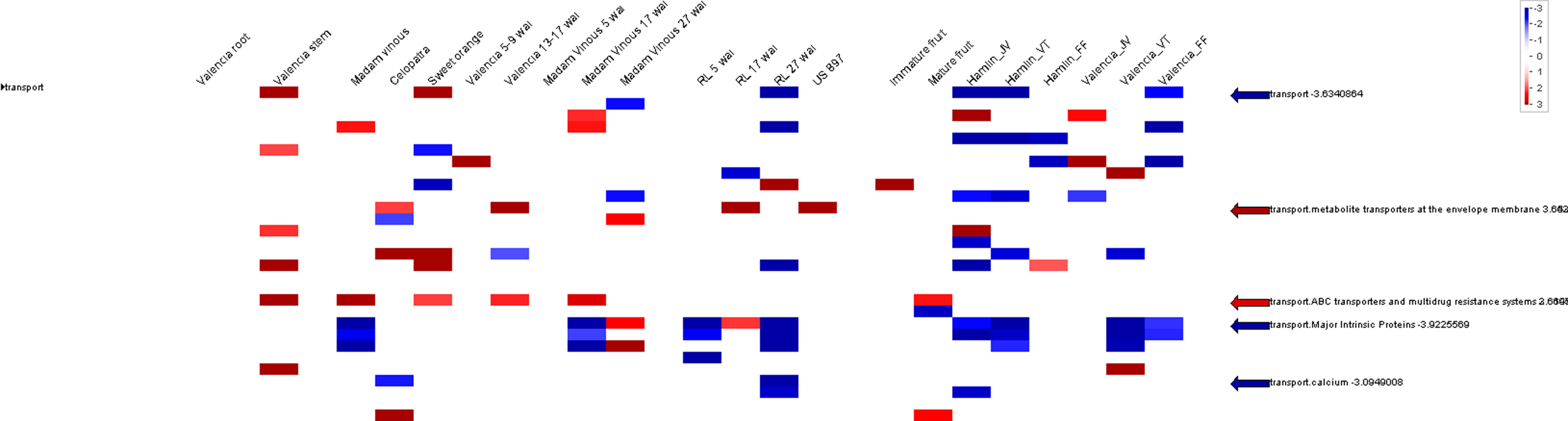

Supplement: Additional file 8: Figure S6. — PageMan display of coordinated changes of “stress” gene categories in 22 data set. [file 12870_2015_568_MOESM8_ESM.jpg]

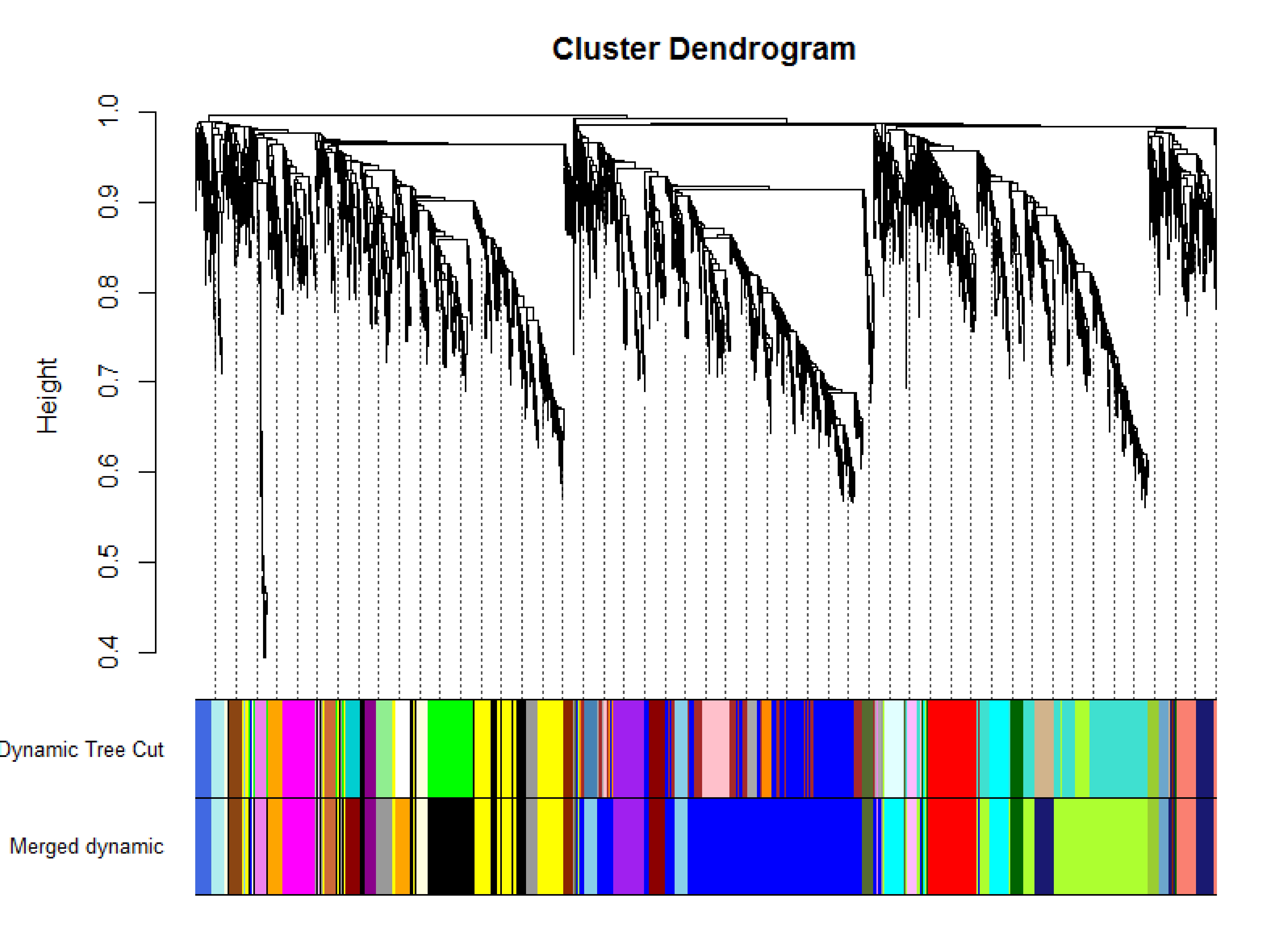

Supplement: Additional file 12: Figure S7. — Dendrogram for co-expressed modules. Dendrogram showing co-expressed modules before and after merging using the WGCNA package. [file 12870_2015_568_MOESM12_ESM.jpg]

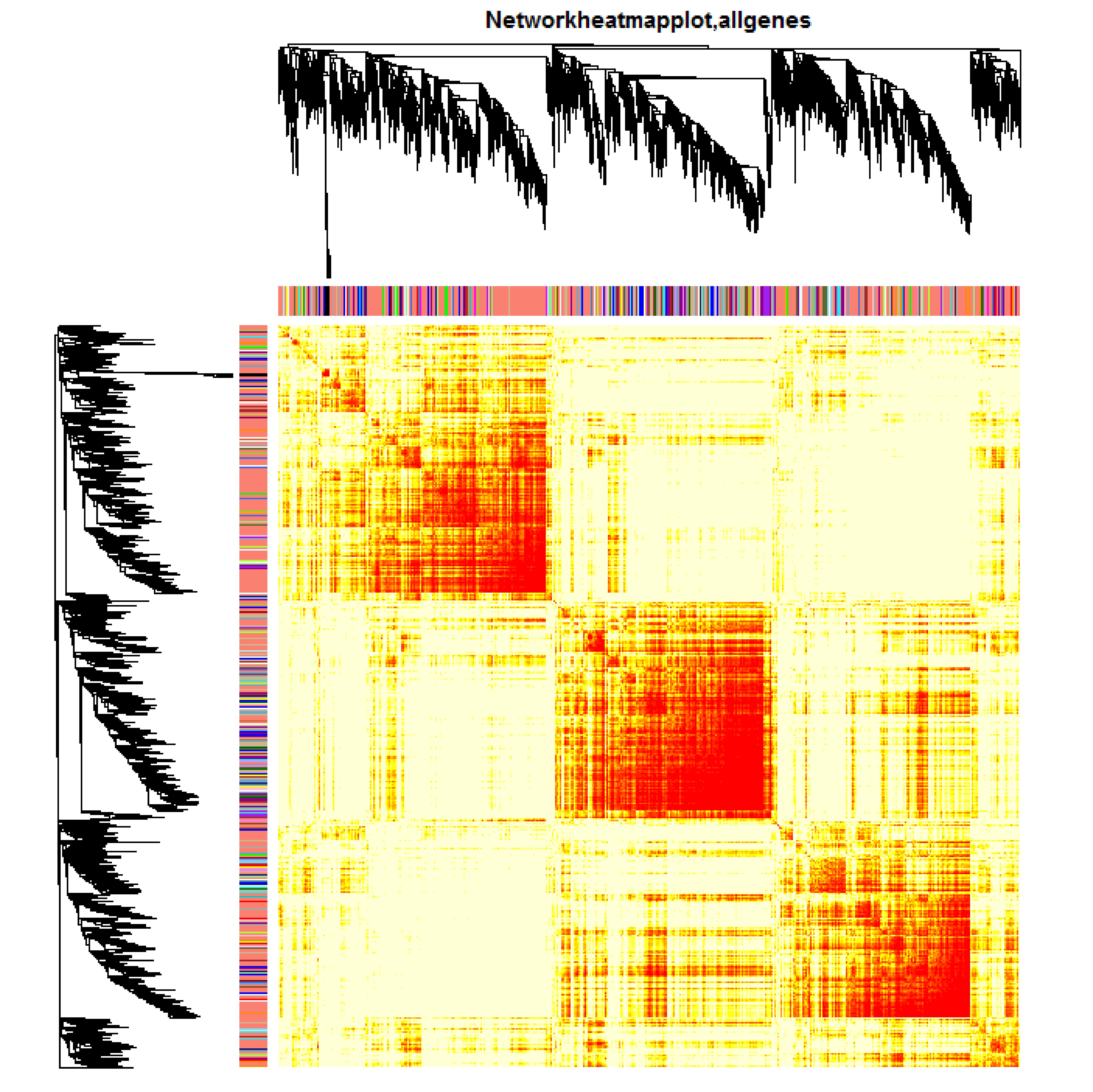

Supplement: Additional file 13: Figure S8. — Network Heatmap plot for co-expressed modules. Heatmap view of topological overlap values in different modules. Red color shows highly co-expressed probe sets forming a module. [file 12870_2015_568_MOESM13_ESM.jpg]
